# Biowaste‐Derived Triboelectric Nanogenerators for Emerging Bioelectronics

**DOI:** 10.1002/advs.202405666

**Published:** 2024-09-09

**Authors:** Abhisikta Bhaduri, Tae‐Jun Ha

**Affiliations:** ^1^ Dept. of Electronic Materials Engineering Kwangwoon University Seoul 01897 Republic of Korea

**Keywords:** biocompatibility, bioelectronics, biowaste, energy harvesting, triboelectric nanogenerator

## Abstract

Triboelectric nanogenerators (TENGs) combine contact electrification and electrostatic induction effects to convert waste mechanical energy into electrical energy. As conventional devices contribute to electronic waste, TENGs based on ecofriendly and biocompatible materials have been developed for various energy applications. Owing to the abundance, accessibility, low cost, and biodegradability of biowaste (BW), recycling these materials has gained considerable attention as a green approach for fabricating TENGs. This review provides a detailed overview of BW materials, processing techniques for BW‐based TENGs (BW‐TENGs), and potential applications of BW‐TENGs in emerging bioelectronics. In particular, recent progress in material design, fabrication methods, and biomechanical and environmental energy‐harvesting performance is discussed. This review is aimed at promoting the continued development of BW‐TENGs and their adoption for sustainable energy‐harvesting applications in the field of bioelectronics.

## Graphical abstract

This review provides a comprehensive summary of studies on biowaste (BW) materials for use in triboelectric nanogenerators (TENGs), with a focus on processing methods, energy‐harvesting performance, and emerging bioelectronic applications. In addition to discussing the current state of research, challenges, limitations, and opportunities in this field are highlighted to guide future work on BW‐TENG technologies.

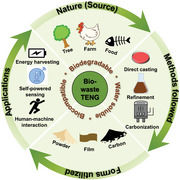

## 1 Introduction

Triboelectric nanogenerators (TENGs) harvest mechanical energy from simple body movements through frictional effects. Owing to the convenience, efficiency, and broad applicability of TENGs, the development of these devices has expanded to incorporate other natural phenomena, such as wind, flowing water, and rain. TENGs are typically employed in small electronic and optoelectronic devices, medical equipment, robotics, and self‐powered electronics.^[^ ^1^ ^]^ However, as these devices commonly consist of materials that are difficult to biodegrade and discharge toxic substances upon degradation,^[^ ^2^ ^]^ TENGs contribute to electronic waste (e‐waste).

Rapid progress in mechanization and technology has sharply increased global e‐waste production. As e‐waste now accounts for a substantial share of solid waste, the numerous non‐biocompatible and hazardous components in e‐waste pose a critical threat to the environment and human health. To limit e‐waste and the associated hazards, biodegradable materials have received increasing interest for the development of environment‐friendly electronics.^[^ ^3^ ^]^ The incorporation of natural, nontoxic, and biocompatible triboelectric components into smart electronics could contribute to the creation of a next‐generation green society.

Natural materials are discarded daily on a large scale. As biowaste (BW) is abundant in common biocompatible materials including cellulose, lignin, chitosan, gelatin, and collagen, which can participate in triboelectric production,^[^ ^4^ ^]^ such has been considered for TENG development. The recycling of BW as triboelectric layers that harvest mechanical energy does not require complicated methods. Thus, the large‐scale production and commercialization of next‐generation BW‐based TENGs (referred to as BW‐TENGs hereafter) provides an effective approach for utilizing BW while actively reducing e‐waste toward the realization of a pollution‐free society. BW‐TENGs have attracted significant attention in the field of next‐generation energy harvesting technologies for various electronics including sensors and displays toward achieving the goal of net‐zero emissions.^[^ ^6^ ^]^ Many studies on BW‐TENGs have primarily focused on mechanical and biomechanical energy harvesting. Owing to their nontoxic and biocompatible nature, BW‐TENGs can be applied in real‐time healthcare and biomedical devices to harvest energy from limb movements, pulses, and heartbeats. Advantageously, these devices can be easily absorbed by the body without negative effects, eliminating the need for surgical removal.^[^ ^5^ ^]^ Since 2021, the applicability of BW‐TENGs in other fields, such as human–machine interfaces (HMIs) and self‐powered bioelectronics, has also been explored.

Notably, BW‐TENGs produce a wide range of output voltages, from a few volts to thousands of volts, which facilitates integration with a variety of devices. Moreover, these energy‐harvesting components offer impressive sustainability, addressing environmental issues including the increasing production of BW and e‐waste as well as the exhaustion of nonrenewable energy sources. However, as BW‐TENGs are a relatively new approach, many aspects of these systems, such as output optimization and application scope, require further investigation. Although TENGs based on active materials such as bioresorbable materials,^[^ ^6^ ^]^ degradable materials,^[^ ^7^ ^]^ cellulose,^[^ ^8^ ^]^ textiles,^[^ ^9^ ^]^ and polymers^[^ ^10^ ^]^ have been demonstrated, this is the first review to provide a systematic overview of BW‐TENGs.

Herein, we discuss recent progress on BW‐TENGs, with a focus on the use of different types of BW, performance characteristics, and applications as triboelectric components for bioenergy materials, biotechnologies, and bioelectronics. Specifically, Section 1 introduces BW‐TENGs and the need for biocompatible and biodegradable materials in current electronic applications. Section 2 discusses biocompatible and biodegradable types of BW as sustainable bioenergy materials, including the origin of their triboelectric properties, and BW processing methods for preparing TENGs. Section 3 reviews the biomechanical and environmental energy‐harvesting performance of various BW‐TENGs. Section 4 outlines emerging bioelectronics based on BW‐TENGs for next‐generation biotechnologies. Finally, Section 5 discusses challenges, limitations, and opportunities for further advancing the development of BW‐TENGs. In addition to highlighting the suitability of BW‐TENGs for commercialization owing to the abundance of their constituent materials and ability to reduce environmental pollution, this review is anticipated to inspire future research on BW‐TENGs and their applicability in various fields.

## 2 BW Materials and Derivation Methods for TENG Fabrication

### 2.1 BW Materials

**Figure 1:**
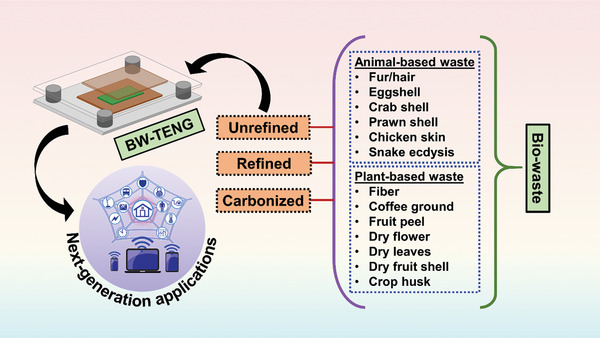
Schematic of BW materials used for TENG fabrication.

Waste materials can be categorized according to various factors, including the state of matter, environmental impact, degradability, and source. BW materials are defined as materials originating from animal or plant sources that easily degrade in the environment owing to biotic (e.g., fungi, bacteria, and plants) and abiotic (e.g., temperature, humidity, and pH) factors. BW materials are widely found in domestic/municipal and agricultural waste. Owing to their biocompatibility, abundance, easy accessibility, cost‐effectiveness, and biodegradability, BW materials have been widely applied in supercapacitors, tissue engineering, and functional devices.^[^ ^11^, ^12^ ^]^ Both animal‐ and plant‐based TENGs have been used to fabricate TENGs, as outlined in **Figure** **1**. The intrinsic morphologies of certain BW materials allow them to be directly employed for specialized surface‐related applications without additional treatment.^[^ ^13^ ^]^ Refined BW products, such as gelatin, chitosan, and cellulose, are abundant and ecofriendly materials that are suitable for numerous applications.^[^ ^14^ ^]^ However, the high hydrogen bond content of BW makes these materials sensitive to humidity and environmental factors. To overcome this issue, BW materials have been functionalized, combined with other materials, or carbonized. Carbonization can increase conductivity, flexibility, and hydrophobicity, thereby enhancing the energy conversion efficiency and stability of BW materials under various environmental conditions.^[^ ^15^ ^]^

### 2.2 Advantages of BW Materials for TENGs

#### 2.2.1 Self‐Powered Sensors

For self‐powered sensing applications, an active sensing material needs to be well‐interacted with the target entities besides being tribo‐active. The cellulose‐enriched BW‐materials like fruit and vegetable peels can interact well with the humidity and gases, by facilitating the reaction between the gas/water molecules and BW‐material.^[^ ^14^ ^]^

#### 2.2.2 Environmental Energy Harvesting

To harvest mechanical energy from the environmental sources like wind breeze and water flow, rotary‐based BW‐TENGs can be used where a specially designed rotor operates over a stator under the influence of wind breeze and water flow.^[^ ^12^ ^]^ As a result of friction between the stator and rotor, the triboelectric output can be obtained. To fabricate these, robust frictional layers are needed, which can withstand environmental conditions and have high durability over long‐term periods. Soft fibers like discarded animal fur can be promising for preparation of soft‐contact rotary‐based BW‐TENGs that provide excellent resilience over long‐term use.^[^ ^8^ ^]^ Moreover, the keratin of the fibers provides high tribo‐output owing to the presence of abundant electron‐donating functional groups.^[^ ^8^ ^]^

#### 2.2.3 Wearable Sensing Applications

For wearable sensing applications, the key feature is flexibility and stretchability. Most of the plant fibers own interconnected complex inherent microstructures that provide high flexibility and stretchability without any further modifications. Hence, the plant fibers such as Juncus effusus fibers, loofah, and coffee waste have been widely used in these applications.^[^ ^12^ ^]^ Besides these, other BW materials including flower extract, collagen, gelatin, and chitosan can be modified to hold high flexibility by dispersing them in the suitable solution, which can be used for wearable sensing applications in monitoring physiological sensing owing to their high flexibility and biocompatibility.^[^ ^2^ ^]^

#### 2.2.4 High Triboelectric Output and Electrical Conductivity

For high triboelectric performance, it is crucial to choose suitable materials with strong tendency of electron gaining/loosing. The cellulose and keratin‐based BW‐TENGs are proven to be the one of the promising candidates in terms of generating high triboelectric output.^[^ ^16^ ^]^ The BW material is the abundant source of functional carbon‐based groups, which can produce pure carbonaceous materials upon heat treatments. The highly conductive carbonized BWs can be either used as highly conducting electrodes, or added into the active material, to enhance the triboelectric performance.^[^ ^8^ ^]^

#### 2.2.5 Efficiency of BW‐TENGs

The efficiency of a TENG depends on its ability to convert mechanical energy into electrical energy. High‐efficiency TENGs, in which the energy‐harvesting capability is maximized, have potential for diverse applications. BW‐TENGs, which exhibit triboelectricity originating from the inherent structures of the BW materials, generally offer a diverse range of output powers. In a few reports, BW has been combined with other materials to increase the energy conversion efficiency; however, the approach requires further investigations. The development of engineered structures represented a major breakthrough. Rotary TENGs were prepared using soft dog fur, rabbit fur, or wool stuck to a rotor plate.^[^ ^16^ ^]^ The choice of rabbit fur combined with an engineered structure resulted in high energy conversion.^[^ ^16^ ^]^ Depending on material characteristics, BW‐TENGs often exhibit high power densities, even in the basic contact‐separation (CS) mode.^[^ ^17^, ^18^, ^19^ ^]^ **Table** **1** shows a comparison of the output powers generated by highly efficient BW‐TENGs (in bold) with those of other conventional systems. In addition to being easy and inexpensive to fabricate, the BW‐TENGs provide outputs that are comparable to or even better than those of the conventional devices. The high outputs of the BW‐TENGs can be attributed to the presence of abundant electron‐donating functional groups, inherent stress‐induced polarization, and unique microstructures, as discussed in detail in Section 2.4.

**Table 1:** Comparison of various TENG outputs, including power/power density.

No.	Triboelectric materials used in TENG			Triboelectric outputs			Ref.
	Type of materials used (waste or not)	Material 1	Material 2	Voltage	Current/Current density	Power/Power density	
1	Not waste	PVDF‐TrFE/MXene	Nylon	270 V	140 mA m^−2^	4.02 W m^−2^	[235]
2	Not waste	2D‐hexagonal boron nitride nanosheets printed on Mylar substrate	Polyvinylpyrrolidone (PVP) printed on Mylar substrate	800 V	0.78 mA m^−2^	1.36 W m^−2^	[236]
3	Not waste	Natural rubber‐TiO_2_	PTFE	113 V	9.8 µA	237 mW m^−2^	[237]
4	Medical waste (non‐bio)	X‐ray sheet	Silicone	201 V	62.8 µA	1.39 W m^−2^	[238]
5	Non‐bio waste	Sock cloth single piece	PTFE	267 V	3.4 µA	225.6 µW	[239]
6	Non‐bio waste	Graphite from pencil/PDMS	Nylon	187 V	28 µA	1.2 mW cm^−2^	[240]
7	Non‐bio waste	Cigarette filters	PDMS	96.63 V	9.37 µA	413 mW m^−2^	[241]
8	Not waste	Hydrogel wrapped with VHB films	PTFE	75 V	0.6 µA	260 mW m^−2^	[242]
9	Non‐bio waste	Polyvinyl chloride (PVC) plastic layer	Nylon (PA) plastic layer	35.7 V	5.85 µA	152.6 mW m^−2^	[243]
10	Non‐bio waste	Polypropylene from used face mask	Copper	200 V	0.29 mA m^2^	71.16 mW m^−2^	[244]
11	Non‐bio waste	Laboratory waste plastic‐glass	PET	185 V	1.25 µA	81 mW m^−2^	[245]
12	Not waste	Cd‐MOF/PDMS	Copper	193.4 V	0.86 µA	0.124 W m^−2^	[246]
13	Not waste	PVC gel	Nylon	24.7 V	0.83 µA	8.7 µW cm^−2^	[247]
14	Non‐bio waste	Al foil from food package	PET	4 V	–	40 nW cm^−2^	[248]
15	Not waste	BaTiO_3_/PVDF	Nylon	160 V	6.2 µA	225.6 mW m^−2^	[249]
16	Bio‐waste	Lignin fiber mat with carbonized lignin fiber mat as electrodes	Nitrocellulose fiber mat	232 V	17 mA m^2^	1.6 W m^−2^	[60]
17	Bio‐waste	Cellulose nanofiber from sugarcane bagasse + Activated carbon + Natural rubber	PTFE	137 V	12.1 µA	2.74 W m^−2^	[37]
18	Bio‐waste	Chicken skin in single piece	Kapton	123 V	20 µA	0.2 mW cm^−2^	[108]
19	Bio‐waste	Single piece of wasted printed paper	FEP	774 V	3.92 mA	43.5 W m^−2^	[17]
20	Bio‐waste	Half‐cell film from monolayer cell film of Leek skin	Other half of the cell film	182 V	0.83 mA m^2^	35 W m^−2^	[18]
21	Bio‐waste	Agricultural plant debris fiber	Greenhouse film	2600 V	90 µA	1.24 W m^−2^	[77]
22	Bio‐waste	Milled waste tea leaves	PTFE	792 V	42.8 µA	488.88 µW cm^−2^	[250]
23	Bio‐waste	Corn husk small pieces, Coconut coir small pieces	2D‐gC_3_N_4_	600 V, ≈600 V	0.79 mA, 11.47 mA	32.75 mW cm^−2^ 495 mW cm^−2^	[19]
24	Bio‐waste	Human hair powder + polymeric matrix	Perfluoroalkoxy (PFA)	296 V	37.6 µA	0.6 mW cm^−2^	[146]
25	Bio‐waste	Numerous rabbit hair strands stuck on rotor plates	PTFE	≈10 000 V (extracted from figure)	≈40 µA (extracted from figure)	1200 mW	[207]

### 2.3 BW Derivation Methods

BW materials demonstrate substantial advantages in biotechnological purposes owing to their unique features including biocompatible, biodegradable, abundant, cost‐effective, and easily accessible merits. Untreated BWs retain their inherent microstructural characteristics, which is beneficial for bioelectronic applications through surface‐related interactions, without the need of any chemical refinements. Besides their regular constituents like cellulose, lignin, and hemicellulose, untreated BWs possess unique polymeric constituents that affect their electronic properties. On the other hand, chemically refining or treating the BWs and other nature‐derived materials can induce carbon‐neutral biopolymers, such as cellulose, lignin, chitosan, and gelatin, which have been widely utilized in various biomedical applications. The treated BWs and their derivatives can be used extensively for flexible bioelectronics owing to their flexible, lightweight, and progressively functional properties.

BWs can be differentiated by the terms of the forms they are employed for. The untreated BWs which do not follow any chemical post‐treatments, can offer simple and inexpensive TENG fabrication. In general, untreated BWs are directly adhered onto the TENG electrode. For better dispersion, the BW extracts added in polymeric media are used as an active material. The microstructures of the BWs contain unwanted substances such as fiber lining, dirt, ashes, wax, lipid, etc., limiting their physical and chemical properties. In those cases, the BWs which are chemically refined to avail the corresponding biopolymers in their purest form deliver stable and durable TENG performance. In addition, it is favorable to combine the biopolymers with other materials where their output characteristics can be modified. The untreated BWs are generally vulnerable to humidity, which can easily be removed in the treated BWs via further chemical treatments. On the other hand, carbon is generally used for TENGs with other materials to improve triboelectrification and charge transfer. BWs being the abundant source of carbon, are often sintered following various methods as cost‐effective approaches for TENG fabrication. The BWs with interconnected fiber‐like structures are directly sintered to reveal interconnected flexible carbon fibers, which can be useful for self‐powered strain sensing.

Till date, untreated BWs have been mostly employed for TENGs to investigate their electrical energy generation ability owing to their coherent structures and chemical properties. On the other hand, very few carbonized and treated BW‐TENGs have been reported. As their triboelectric characteristics depend heavily on the constituent biopolymers and functional groups, we differentiated the energy harvesting section in terms of their main components.

#### 2.3.1 Untreated BW Materials

Plant‐based TENGs can be prepared by employing BW from different plant parts directly in TENGs without chemical treatment.^[^ ^4^ ^]^ Untreated BW materials are easily available, biodegradable, environmentally friendly, and nontoxic. Furthermore, this fabrication method is simple and does not require costly refinement processes. Untreated BW‐TENGs are produced by either sticking materials directly onto an electrode or using a powdered dispersion in a polymeric medium to prepare a TENG layer that imitates the source characteristics.^[^ ^20^ ^]^

**Figure 2:**
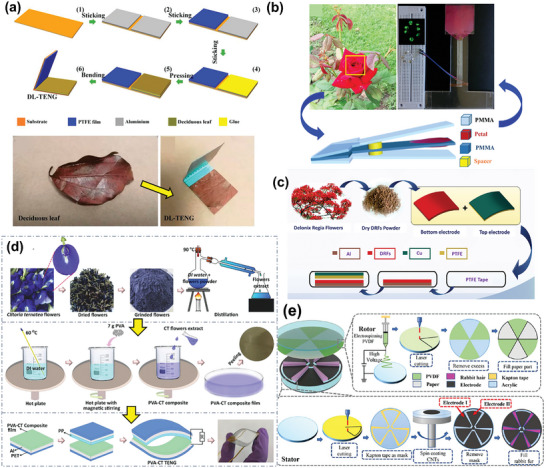
Schematics illustrating the fabrication of untreated BW‐TENGs using a) a piece of fallen deciduous leaf. Reproduced with permission.^[^ ^22^ ^]^ Copyright 2021, MDPI; b) rose petals;^[^ ^23^ ^]^ c) *Delonix regia* flower (DRF) powder adhered to an electrode. Reproduced with permission.^[^ ^24^ ^]^ Copyright 2022, Wiley‐VCH; d) *Clitoria ternatea* (CT) flower extract distributed in a PVA matrix. Reproduced with permission.^[^ ^25^ ^]^ Copyright 2023, Elsevier; and e) rabbit fur stuck a rotor in a rotary TENG. Reproduced with permission.^[^ ^32^ ^]^ Copyright 2023, IOP publishing.

Plant leaves are the most widely utilized plant‐based biomaterials in biocompatible TENGs.^[^ ^21^ ^]^ A piece of fallen deciduous leaf (DL, thickness of ≈300 µm) was directly pasted on an Al foil to fabricate a TENG with PTFE as a second dielectric layer in the CS mode (**Figure** **2**a).^[^ ^22^ ^]^ Under continuous vibration, this BW‐TENG‐generated electrical signals of 150 V, 4.2 µA, and 38.4 nC. Although moisture adsorption by the DL reduced the electrical output in humid environments, this issue was successfully addressed by introducing a packaged structure to provide moisture resistance. The triboelectric output was further enhanced by stacking multiple TENGs in parallel. However, the long‐term stability of this device was not tested owing to the low durability of the TENG active material, that is, the DL is brittle and can easily fall off under continuous tapping.

Flower petals are often used in TENG fabrication because they have unique microstructures with large specific surface areas and contain distinct organic complexes with electron‐donating functional groups. Red rose petals were attached to a single electrode for triboelectrification (Figure 2b). The large effective surface area provided by the C chains of the epicuticular wax on the rose petals enhanced the TENG performance.^[^ ^23^ ^]^ However, the operation of this TENG was limited by withering of the fresh petals. To avoid this issue, sundried *Delonix regia* flowers were crushed into a fine powder using a mortar and pestle, sieved, and directly attached to an Al electrode with a PTFE layer attached to the other electrode to prepare a BW‐TENG with a long operating time (Figure 2c).^[^ ^24^ ^]^ Field‐emission scanning electron microscopy revealed a random size distribution of flower particles, which resulted in slight variations in the TENG output. Furthermore, the device had low stability, with film wear and performance deterioration occurring after 18 000 CS cycles. These limitations were overcome using a coarse powder of *Clitoria ternatea* flowers soaked in distilled water and distilled at 90 °C. Subsequently, the extract was added to a PVA solution, magnetically stirred, and sonicated to achieve a uniform dispersion (Figure 2d).^[^ ^25^ ^]^ The effective distribution of the powder phase in the polymer matrix was confirmed using scanning electron microscopy and powder X‐ray diffraction. This BW‐TENG exhibited high durability and stability after energy harvesting for over 8 months.

Food waste is an important plant‐based source for triboelectricity generation. The thin skins of tomatoes,^[^ ^26^ ^]^ onions, and garlic^[^ ^27^ ^]^ were directly adhered to the electrode, whereas the hard shells of dry fruits^[^ ^28^, ^29^ ^]^ in powder form were used as the active TENG layer. Owing to their differences in chemical composition and surface morphology, the half‐cell skins of *Allium* plants can be used as hetero‐triboelectric materials for TENG development.^[^ ^18^ ^]^

Rabbit fur is readily available and soft with a porous fiber‐like structure that mainly consists of the electron‐donating protein keratin, a triboelectric biomaterial. The fibrous structure provides a large surface area, whereas the softness ensures satisfactory friction at high‐speed rotation without abrasion.^[^ ^30^, ^31^ ^]^ A rotary TENG with clean rabbit fur stuck to the rotor plate as a soft frictional layer (Figure 2e) generated ∼350% higher triboelectric output than the direct‐contact configuration and remained stable for up to 100 000 cycles.^[^ ^32^ ^]^

#### 2.3.2 Treated BW Materials

**Figure 3:**
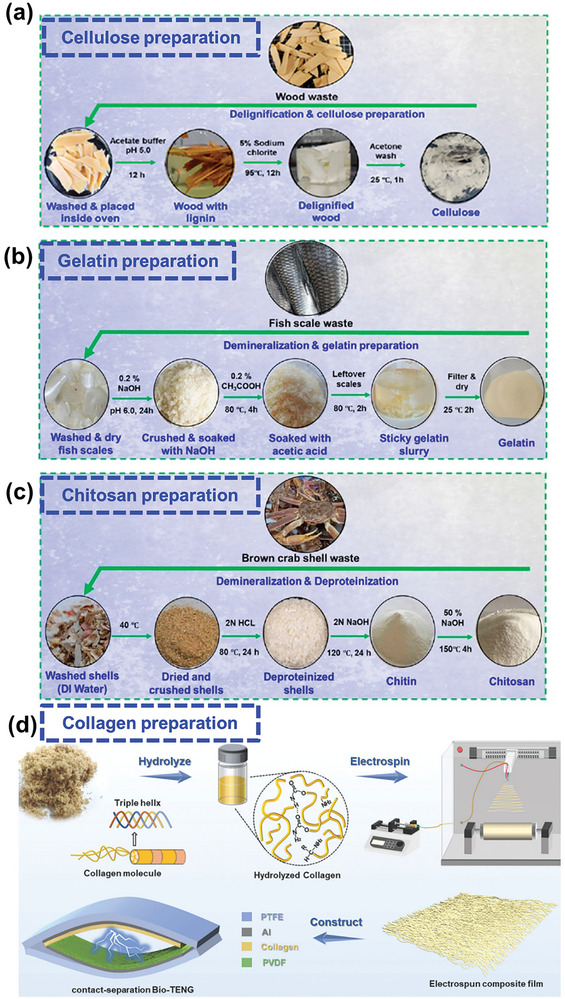
Schematics of chemical refinement processes for preparing a) cellulose from wood waste. Reproduced with permission.^[^ ^35^ ^]^ Copyright 2023, American Chemical Society; b) gelatin from fish scale waste. Reproduced with permission.^[^ ^35^ ^]^ Copyright 2023, American Chemical Society; c) chitosan from crab shell waste. Reproduced with permission.^[^ ^35^ ^]^ Copyright 2023, American Chemical Society; and d) collagen from tanned leather shavings for TENG fabrication. Reproduced with permission.^[^ ^46^ ^]^ Copyright 2022, Royal Society of Chemistry.

The exteriors and microstructures of BW materials are often covered with unwanted substances, such as dirt, ash, wax, and lipids, which influence their physical and chemical properties.^[^ ^33^ ^]^ Hence, chemical treatments are frequently used to remove these substances and obtain the pure constituent fibers. Chemically refined fiber surfaces exhibit increased porosity and roughness, thereby promoting advanced triboelectrification.^[^ ^34^ ^]^ Moreover, the chemical refinement of plant‐based waste produces fibrous cellulose. One of the simplest methods for cellulose extraction is the bleaching of waste wood pieces in acetate buffer, followed by high‐temperature hydrothermal lignin removal in a sodium chlorite solution (**Figure** **3**a).^[^ ^35^ ^]^ Cellulose nanofibers (CNFs) retain excellent mechanical characteristics, such as high strength, high rigidity, low density, and biocompatibility as well as being naturally biodegradable and suitable for chemical modification. When used as a triboelectric layer, CNFs produce power densities of up to 300 W m^−2^.^[^ ^36^ ^]^

CNF extraction is also easily achieved using alkali treatment. Specifically, immersion in an alkaline solution removes most lipids, waxes, lignin, and hemicellulose from the target material. For example, discarded sugarcane bagasse was treated in NaOH solution at 90 °C for 4 h, followed by bleaching in alkaline peroxide (H_2_O_2_:NaOH = 4:1), resulting in fine CNFs.^[^ ^37^ ^]^ A composite film prepared using HCl‐hydrolyzed CNFs, natural rubber, and activated C generated a TENG power density of ≈2.74 W m^−2^. However, this process did not yield well‐separated CNFs.

The 2,2,6,6‐tetramethylpiperidine‐1‐oxyl radical (TEMPO) oxidation method was developed as a unique method for preparing individual CNFs.^[^ ^38^ ^]^ Here, an aqueous wood pulp suspension was treated with TEMPO, NaBr, and NaClO while maintaining the pH using NaOH.^[^ ^39^ ^]^ This process facilitated the exchange of –OH groups on the CNF surface with carboxylates, rendering negatively charged CNFs. The strong repulsion generated within the material promoted nanofiber separation. In particular, ultrasonication produced well‐separated CNFs with high length‐to‐radius ratios.^[^ ^38^ ^]^

Cellulose nanocrystals (CNCs) are the building blocks of commercially available paper.^[^ ^40^ ^]^ The extraction of CNCs from discarded printing paper requires a slightly different process. Paper pulp was first bleached in NaOH and NaClO solution to remove ink, lignin, and hemicellulose, resulting in microcrystalline cellulose, the amorphous section of which were eliminated using H_2_SO_4_ solution. Finally, pure CNCs were obtained by dialysis.^[^ ^41^ ^]^ Advantageously, these CNCs maintained strong bonding with surrounding materials in composites, making this material a suitable filler for preparing biocompatible and wearable TENGs.^[^ ^41^ ^]^

Animal‐based waste materials are mainly composed of collagen, gelatin, and the polysaccharide chitin, which can be extracted using simple chemical treatment methods.^[^ ^42^ ^]^ Gelatin is abundant in fish scales, which are discarded daily as food waste. A gelatin slurry was prepared by dispersing precleaned and dried fish scales in NaOH solution to remove unwanted complexes and then soaking in an acidic medium (Figure 3b).^[^ ^35^ ^]^ Following filtration, pure gelatin was obtained, which could be employed in mechanical energy harvesting.

As a hydrolyzed derivative of chitin, which is widely found in crustacean shells, chitosan can be isolated from food waste for use in BW‐TENGs (Figure 3c).^[^ ^35^ ^]^ Specifically, discarded shells were ultrasonically cleaned, dried, and treated with a heated HCl solution for demineralization to remove calcium carbonate and other impurities. Subsequently, the demineralized product was repeatedly immersed in a heated NaOH solution to remove proteins. Pure chitin was again treated with a heated NaOH solution to produce pure chitosan via deacetylation. Using 2 wt.% BW‐derived cellulose, chitosan, or gelatin dispersed in PVA solution, BW‐TENGs were fabricated in single‐electrode (SE) mode with consumable Ag leaf as the electrode. The device with the chitosan‐based composite exhibited better triboelectric characteristics than those with the other two composites. The TENG voltage, current, and power were optimized via compositional engineering, and the highest values of ≈20 V, 200 nA, and 0.45 µW, respectively, were achieved for the 10 wt.% chitosan–PVA sample. The working layer decomposed within 60 s of immersion in phosphate‐buffered saline, confirming the biodegradability of this material.

As an animal‐derived complex polymer, collagen is the main structural protein in various tissues and requires a lengthy extraction process.^[^ ^43^, ^44^ ^]^ In the tannery industry, ∼80% of waste is disposed of in dumping grounds.^[^ ^45^ ^]^ Collagen‐enriched (≈90%) leather shavings from the tannery industry can be refined and hydrolyzed to prepare biocompatible BW‐TENGs (Figure 3d).^[^ ^46^ ^]^ The discarded shavings were cleaned in 1% sodium dodecyl sulfate solution, disinfected with ethanol, sonicated in an ethanolic dispersion, dried, ground, and sifted to obtain fine particles. Subsequently, pure hydrolyzed collagen was obtained by treating the shavings with NaOH and urea solutions for ≈8 h and dialyzing for 2 days. Collagen hydrolysate blended with PVA and 0–10 wt.% Ag nanowires (NWs) was electrospun to form tribo‐positive films. The sample with 8 wt.% Ag NWs generated the highest voltage and charge of 118 V and 52 nC, respectively, against a polyvinylidene fluoride (PVDF) film as the tribo‐negative layer.

#### 2.3.3 Carbonized BW Materials

BW is among the best candidates for carbonized material production owing to its low cost, wide availability, sustainability, versatility, biocompatibility, and renewable sources. Advantageously, the structure of the original BW material is typically maintained after carbonization. Therefore, the final product has a well‐organized and cross‐linked microstructure, which provides excellent ductility, flexibility,^[^ ^47^ ^]^ and thermal conductivity.^[^ ^48^ ^]^ Biomaterials, especially BW, are extremely hydrophilic because of their ability to form numerous hydrogen bonds at the macromolecular level. Upon sintering these materials at high temperatures, hydrophobic functional groups, such as –OH, –CH_2_, –CH_3_, C–O, and C = O, are eliminated, leaving a hydrophobic C structure.^[^ ^49^ ^]^ Using carbonized biomaterials in/as triboelectric layers improves the energy‐harvesting performance. According to the percolation theory, well‐dispersed conducting C in a polymer matrix can induce high dielectric permittivity.^[^ ^50^ ^]^ Consequently, the effective charge density increases, which is advantageous for triboelectrification. Owing to their high electrical conductivity, carbonized materials facilitate the transfer of triboelectric charges between the TENG layer and electrodes.^[^ ^51^, ^52^ ^]^ However, the use of carbonized BW and biomaterials in TENG fabrication is a new research direction that has not yet been well explored.

Organic complexes, including BW materials, can be carbonized using high‐temperature sintering, hydrothermal treatment, and laser‐induced graphitization (LIG). The mechanical, structural, electrical, and chemical properties of the final product can be modified by varying the carbonization conditions. The resulting C/C‐based materials are useful in various applications owing to their high electrical and thermal conductivities, hydrophobicity, flexibility, humidity, and corrosion resistance originating from the presence of extended C structures with various hybridization states.^[^ ^53^ ^]^ During high‐temperature sintering, organic compounds are carbonized or thermally decomposed in a furnace in the presence or absence of a gas environment. This process breaks bigger molecules into smaller ones, leaving pure C after the desorption of unwanted compounds. High‐temperature sintering often decreases the pore size and effective surface area of the final product. To avoid this issue and improve surface activation, biomaterials are often pyrolyzed in two or three steps.^[^ ^54^ ^]^ Notably, banana peels carbonized at high temperatures exhibit extremely porous morphologies and large effective surface areas, which are favorable for energy storage applications.^[^ ^55^ ^]^

**Figure 4:**
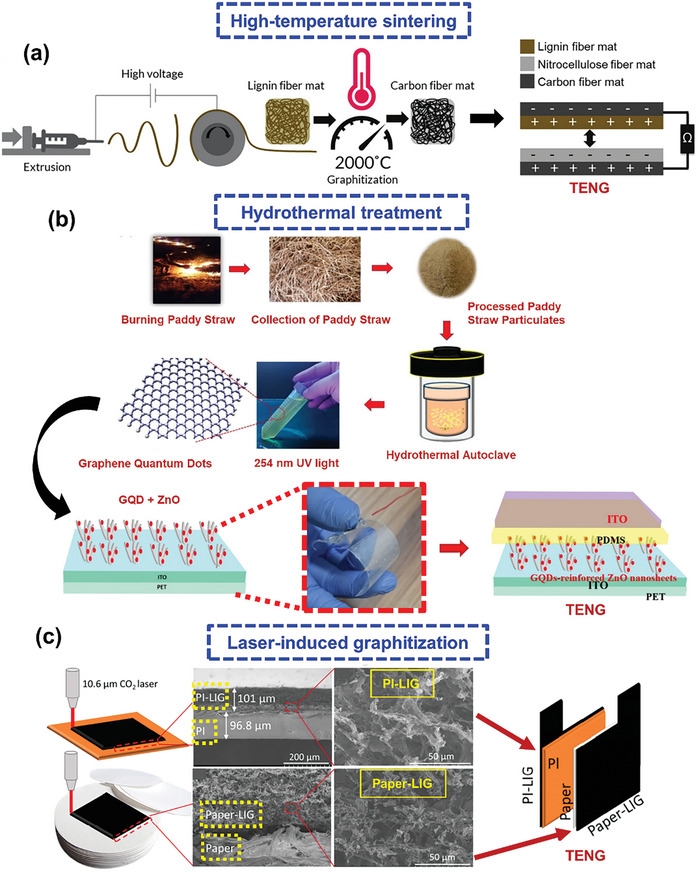
a) High‐temperature sintering for the carbonization of an electrospun lignin fiber mat for TENG fabrication. Reproduced with permission.^[^ ^60^ ^]^ Copyright 2022, Wiley‐VCH. b) Hydrothermal treatment of discarded paddy straw to prepare graphene quantum dots for composite TENG fabrication. Reproduced with permission.^[^ ^62^ ^]^ Copyright 2023, American Chemical Society. c) LIG of PI and paper to fabricate a TENG with built‐in electrodes. Reproduced with permission.^[^ ^72^ ^]^ Copyright 2020, Elsevier.

High‐temperature sintering is also suitable for the facile production of doped C. Doping carbonized materials with other elements, such as N and S, can increase the number of surface active sites, thus increasing reactivity.^[^ ^56^ ^]^ Degreasing cotton soaked in a urea solution at 700 °C under a N_2_ atmosphere produced a highly porous aerogel of N‐doped C fibers.^[^ ^57^ ^]^ Similarly, silver willow blocks carbonized in an Ar environment produced thin‐walled hollow microdimensional C tubes doped with S, P, and N.^[^ ^58^ ^]^ The intercellular layers and lignin were removed from the wood flakes via acid treatment, fermentation, freeze‐drying, and two‐step carbonization to retain the porous cell‐cavity‐like wood structure. Subsequently, this 3D porous carbonized material was employed to fabricate a flexible composite‐based strain sensor and energy harvester.^[^ ^59^ ^]^ Lignin, a byproduct of the paper and pulp industry, is the most abundant biodegradable polymer after cellulose. A thermally stable lignin fiber mat was prepared by dissolving 47 wt.% lignin in dimethyl formamide, electrospinning at 17 kV, and heating.^[^ ^60^ ^]^ The mat was then carbonized in a tube furnace under a N_2_ environment (**Figure** **4**a) to prepare an electrode for a biocompatible TENG. When subjected to mechanical vibration, the biodegradable TENG with lignin and nitrocellulose fiber mats as tribo‐positive and tribo‐negative layers, respectively, produced electrical outputs of 232 V, 17 mA m^−2^, and 1.6 W m^−2^.

The hydrothermal method, also known as wet pyrolysis, is generally employed to convert biomass into homogeneous compact carbon materials using water as a solvent at high temperatures (up to 300 °C) and pressures (typically, 2–6 MPa). The resulting carbon materials retain uniform shapes and sizes with high porosities, surface‐to‐volume ratios, and electrical conductivities.^[^ ^61^ ^]^ Graphene quantum dots were synthesized from a fine powder of discarded paddy straw via a hydrothermal process after treatment in HCl solution (Figure 4b).^[^ ^62^ ^]^ A flexible BW‐TENG was prepared by integrating graphene and ZnO into a PDMS network. Compared to the TENG with ZnO only, the crystalline graphene‐reinforced TENG exhibited a significantly higher dielectric constant and enhanced TENG electrical signals. This enhancement can be ascribed to space‐charge polarization and interfacial polarization in the heterogeneous hybrid system.

Unlike other techniques, LIG involves the spot carbonization of biomaterials using a high‐intensity laser. The laser beam rapidly heats the material surface to temperatures as high as 1000 °C. The biocomplexes at the target site are almost immediately pyrolyzed, thereby transforming into graphene.^[^ ^63^ ^]^ Advantageously, LIG can be used to create pyrolysis patterns without using chemicals. LIG‐carbonized biomaterials have predominantly been investigated for sensors and supercapacitors.^[^ ^64^ ^]^ Moreover, this process has mostly been applied to wood‐based biomaterials, such as paper, cellulose, lignin, and cork. With these materials, LIG is typically performed in an inert environment to avoid ablation.^[^ ^65^ ^]^ The implementation of LIG patterning in open air requires chemical pretreatment of the sample. Recently, grid‐patterned layers of graphitized C were generated on wood using a CO_2_ laser to generate salt‐resistant solar steam.^[^ ^66^ ^]^ This procedure did not include any pretreatment nor an inert environment because the wood surface was first graphitized by laser irradiation at extremely small intervals before inscribing a grid‐like structure by varying the laser depth and interval. Graphene fibers with customizable lengths were prepared by controlling the power density of the incident laser beam.^[^ ^67^ ^]^ The resulting TENG with an output of ≈512 mW m^−2^ was successfully used as a touch sensor.

Paper was the first biomaterial to be graphitized using defocused laser irradiation, whereby graphitized patterns were fabricated on different types of paper.^[^ ^68^ ^]^ Commercial fire retardants,^[^ ^69^ ^]^ sodium borate‐^[^ ^70^ ^]^ and phosphate‐based additives,^[^ ^71^ ^]^ and other chemical complexes are generally used to pretreat the paper samples. The conventional metal electrodes of a TENG were replaced with electrodes prepared by subjecting polyimide (PI) and paper to LIG.^[^ ^72^ ^]^ To achieve graphitization, a CO_2_ laser (wavelength: 10.6 µm, power: 8.1 W) was used to irradiate the untreated PI film once and a commercial‐fire‐retardant‐treated piece of paper twice. The fabricated TENG and morphologies of the LIG‐produced components are shown in Figure 4c. The as‐prepared TENG with crystalline graphene electrodes generated a peak‐to‐peak voltage of ≈625 V and a current of ≈20 mA m^−2^. Electrode formation directly on the TENG layers resulted in extremely close contact between the TENG and electrode surfaces, thereby inhibiting charge loss from the TENG layer to the electrodes after triboelectrification. Consequently, the LIG‐based TENG generated an output power of ≈2.25 W m^−2^, which is 150% greater than that generated by the corresponding TENG with Al electrodes.

### 2.4 TENG Mechanism

#### 2.4.1 Basic Working Principle of TENGs

Irrespective of their structures, all TENGs can efficiently convert mechanical energy into electrical signals through the combined effects of contact electrification (CE) and electrostatic induction. CE is defined as the generation of charges with opposing polarities at the surfaces of two materials with different electronegativities when they are separated immediately after being brought into contact with each other.^[^ ^73^ ^]^ CE can arise at the interface between any two phases, including solid–solid, liquid–liquid, gas–gas, solid–liquid, solid–gas, and liquid–gas interfaces.^[^ ^74^ ^]^

**Figure 5:**
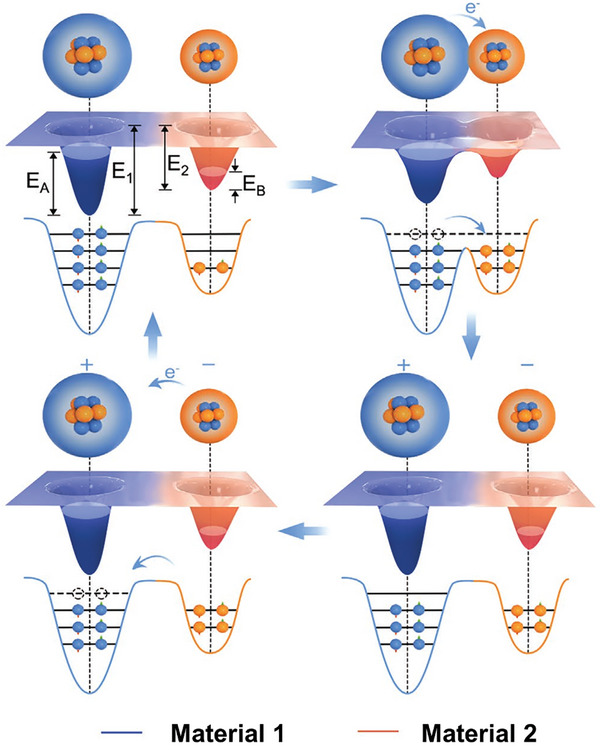
Working mechanism of TENGs depicted using an electron cloud–potential well model.^[^ ^77^ ^]^

At solid–solid interfaces, the CE mechanism has been found to mainly arise from electron transfer.^[^ ^75^ ^]^ Similar behavior has been observed experimentally for the CE phenomenon at a metal–dielectric solid interface. Subsequently, an electron cloud–potential well model was developed to explain the CE mechanism in almost all types of materials,^[^ ^76^ ^]^ as shown schematically in **Figure** **5**.^[^ ^77^ ^]^ Step I shows the electron clouds of materials 1 and 2 when there is no contact between the materials. In this state, the electrons of each atom reside in their original orbitals. Furthermore, the electron clouds are entirely separated from each other owing to the presence of a high interatomic potential barrier between the two materials. As the materials approach atomic‐scale contact with each other, the potential barrier becomes gradually lower, and the electron clouds begin to overlap. Because of the depressed escape potential, electrons are transferred between the materials until equilibrium is reached. Upon separating the materials, the transferred charges remain on the surface of the other material, causing the materials to be oppositely charged. Post separation, an electric field is induced between the electrodes attached to the TENG because of this electrostatic induction. When a load is connected, the induced potential causes the charges to flow from one electrode to the other via the load, generating an electrical current. After all the surface charges have returned to their original state (Step I), the electron clouds become well separated and the electrical current reaches zero. The continuous repetition of contact and release produces an alternating electrical signal.^[^ ^78^, ^79^ ^]^

#### 2.4.2 Source of Triboelectricity in Natural and BW Materials

BW materials contain numerous electron‐donating carboxyl, amino, amide, and hydroxyl groups, that can participate in triboelectricity production. These functional groups form hydrogen bonds that improve the mechanical properties of the TENG layers.^[^ ^80^ ^]^ Cellulose, chitosan, collagen, gelatin, and keratin are natural piezoelectric materials with high piezoelectric coefficients, even in the absence of an external electric field.^[^ ^81^ ^]^ During triboelectric generation, these biomaterials provide additional surface charges owing to the stress‐induced polarization of their constituents.^[^ ^82^ ^]^

**Figure 6:**
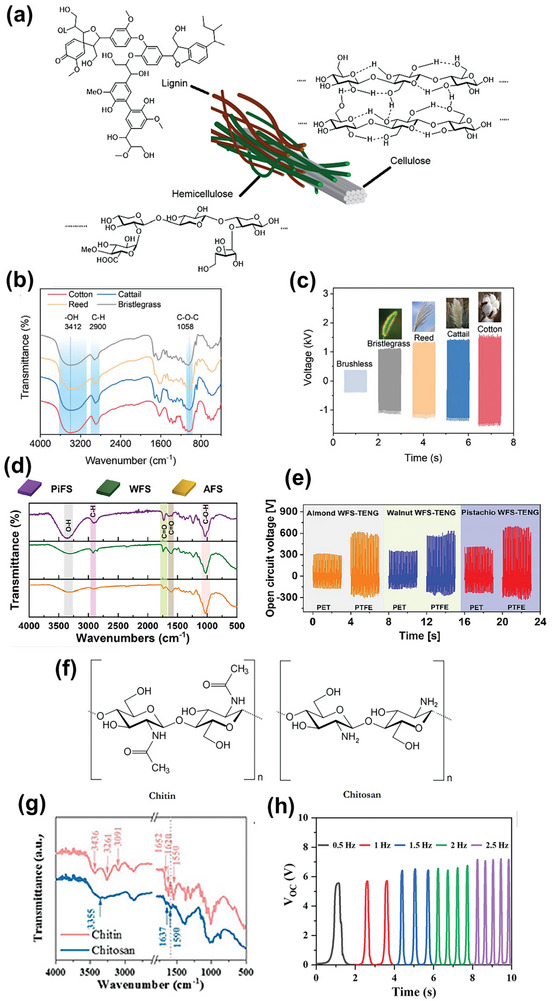
a) Chemical structures of the constituents of lignocellulosic biomass. Reproduced with permission.^[^ ^83^ ^]^ Copyright 2018, Wiley‐VCH. b) FTIR spectra and c) triboelectric voltage generated by various bagasse materials. Reproduced with permission.^[^ ^77^ ^]^ Copyright 2022, Wiley‐VCH. d) FTIR spectra and e) triboelectric voltage generated by dry fruit shells. Reproduced with permission.^[^ ^29^ ^]^ Copyright 2022, Elsevier. f) Chemical structures of chitin and chitosan. Reproduced with permission.^[^ ^94^ ^]^ Copyright 2015, MDPI. g) FTIR spectra and h) triboelectric voltages generated by prawn‐shell‐derived chitosan. Reproduced with permission.^[^ ^96^ ^]^ Copyright 2022, Springer Nature.

Cellulose, an oligosaccharide formed of D‐glucose units linked via β−1,4 glycosidic bonds, is the most abundant biodegradable natural polymer and a basic constituent of every plant, along with hemicellulose and lignin (**Figure** **6**a).^[^ ^83^ ^]^ The ≈1.5 × 10^12^ tons of cellulose derived annually from biomass has potential applications in numerous fields owing to its availability, versatile characteristics, and low cost.^[^ ^84^ ^]^ Moreover, because of its high porosity and large effective surface area, cellulose is an attractive candidate for triboelectric generation. In addition, the high flexibility of cellulose‐based materials is advantageous for fabricating wearable devices.^[^ ^85^ ^]^ Unlike other natural polymers, cellulose does not exist in a monomeric form. Instead, CNFs consisting of cellulose molecular chains connected via hydrogen bonds and van der Waals forces are the basic units of every cellulose‐based material.^[^ ^86^ ^]^ The highly aligned molecular arrangement of these cellulose fibers provides a highly crystalline phase with good mechanical stability, resulting in excellent durability for stress–strain‐related applications.^[^ ^87^ ^]^

Various cellulose‐enriched bagasse materials (cotton, cattail, reed, and bristlegrass), which are considered agricultural waste, have been employed to fabricate BW‐TENGs.^[^ ^77^ ^]^ As shown by the Fourier transform infrared (FTIR) spectra in Figure 6b, all of these samples contain large amounts of –OH groups (≈3412 cm^−1^), which endow tribo‐positive characteristics. In addition to primary and secondary –OH groups, other electron‐donating groups, such as –CH (2928 cm^−1^) and C–O–C (1057 cm^−1^), are predominant on the bagasse surface. Owing to its crystallinity and high content of –OH groups, cellulose has numerous electric dipoles that cause stress‐induced polarization, promoting the triboelectric performance.^[^ ^88^ ^]^ The output voltage of each BW‐TENG (Figure 6c) indicates that each type of bagasse actively participates in triboelectrification. Large amounts of fruit shells are also discarded annually as BW. In addition to cellulose, these shells have high contents of lignin and other O_2_‐rich carbohydrates.^[^ ^89^ ^]^ Figure 6d shows the FTIR spectra of powdered waste pistachio, walnut, and almond shells.^[^ ^29^ ^]^ Along with cellulose‐related peaks, a small peak corresponding to –CH stretching (1462 cm^−1^) is observed owing to the CH_3_ and CH_2_ functional groups in lignin. The differing intensities of the IR bands are related to the different concentrations of cellulose and lignin in each type of fruit shell. The triboelectric properties of TENG layers derived from these fruit shells varied significantly depending on the concentration of electron‐donating complexes (Figure 6e).

The hard shells of insects and marine creatures, such as crabs and prawns, are composed of chitin (poly‐β‐(1,4)‐*N*‐acetyl‐D‐glucosamine), which is a natural polysaccharide.^[^ ^90^ ^]^ Natural chitin exists in three distinct crystalline forms composed of microfibril chains connected in different orientations: α‐chitin with an antiparallel arrangement, β‐chitin with a parallel arrangement, and γ‐chitin with a mixed arrangement.^[^ ^91^ ^]^ Chitosan, derived from chitin via chemical treatment (deacetylation), is highly reactive, biodegradable, and biocompatible with high antibacterial activity,^[^ ^92^ ^]^ making it suitable for medical applications.^[^ ^93^ ^]^ The molecular structure of chitosan is similar to that of cellulose, except that the –OH group at C‐2 is substituted by a –NH_2_ group (Figure 6f),^[^ ^94^ ^]^ which owing to its electron‐donating nature imparts excellent tribo‐positive properties.^[^ ^95^ ^]^ Figure 6g show the FTIR spectra of chitin and chitosan derived from discarded shrimp shells, and Figure 6h shows the triboelectric voltage of the refined chitosan‐based BW‐TENG.^[^ ^96^ ^]^ Chitin and chitosan both exhibit strong peaks corresponding to –OH and –NH_2_ stretching at 3000–3500 cm^−1^ and C═O (amide I) and –NH_2_ (amide II) stretching at 1550–1650 cm^−1^. The observation of a single amide I band and red‐shift in the amide II band in the FTIR spectrum of chitosan confirmed the deacetylation of chitin. Both chitin and chitosan possess noncentrosymmetry that creates natural piezoelectricity^[^ ^97^ ^]^ and contribute to excess charge generation during triboelectrification. The piezoelectric coefficient, d_33_, of commercial chitosan (≈18.6 pC N^−1^ at 300 K) gradually decreases as the temperature increases owing to a loss of noncentrosymmetry.^[^ ^98^ ^]^ In contrast, in prawn‐^[^ ^99^ ^]^ and crab shell‐derived chitin^[^ ^100^ ^]^ have lower d_33_ values of −2 and ≈9.49 pC N^−1^, respectively.

**Figure 7:**
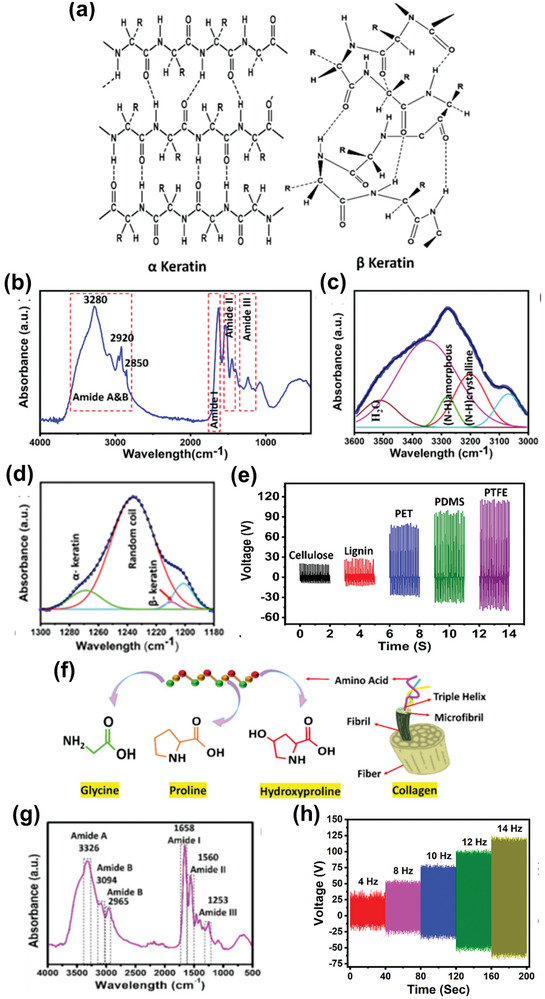
a) Chemical structures of keratin, b) FTIR spectra of keratin‐enriched snake ecdysis and deconvoluted analysis in the regions of c) 3000–3600 and d) 1180—1300 cm^−1^, and e) triboelectric voltages generated snake‐ecdysis‐based TENGs incorporating various materials. Reproduced with permission.^[^ ^103^ ^]^ Copyright 2023, Elsevier. f) Chemical structures of the constituents of collagen, and g) FTIR spectra and h) TENG performance of collagen‐enriched chicken skin. Reproduced with permission.^[^ ^108^ ^]^ Copyright 2023, Springer Nature.

Keratin comprises long chains of α‐helical polypeptides with well‐ordered amino acid units. α‐Keratin, in which a pair of polypeptide chains are twisted around each other to form a helical coil, is found in hair, fur, nails, quills, horns, and skin epidermis. In contrast, β‐keratin, in which the polypeptide chains are attached side‐by‐side to form a sheet‐like structure, is a constituent of scales, claws, beaks, and feathers. As shown by the chemical structures in **Figure** **7**a, α‐ and β‐keratin are both rich in electron‐donating carbonyl groups and O_2_,^[^ ^101^ ^]^ but α‐keratin provides flexibility while β‐keratin provides rigidity. When paired with an electronegative layer, keratin‐based biomaterials generate triboelectricity in response to mechanical vibrations.^[^ ^102^ ^]^

The triboelectric performance of snake‐ecdysis‐based TENGs incorporating different materials as tribo‐negative layers is shown in Figure 7e. Snake ecdysis, which is nontoxic and environmentally friendly, is both flexible owing to the presence of α‐keratin and rigid owing to the presence of β‐keratin. The FTIR spectrum of snake ecdysis shows intense peaks corresponding to carbonyl, hydroxyl, and amino groups (Figure 7b–d), which can participate in triboelectricity generation (Figure 7b–d).^[^ ^103^ ^]^ Multiple absorption peaks ascribed to amide I (C═O stretching), amide II (C–N stretching), and amide III (C–N bending) are observed at 1600–1700, 1500–1580, and 1250–1350 cm^−1^, respectively. These features indicate the presence of α‐keratin, intra‐/intermolecular β‐keratin, and random coils, which are responsible for the natural piezoelectric characteristics of keratin‐based biomaterials. In particular, the α‐helical polypeptide chains of keratin are maintained via hydrogen bonding between O in the C═O groups and H in the amino groups, generating inherent piezoelectricity. Similar patterns were observed in the FTIR spectra of human hair,^[^ ^104^ ^]^ animal fur,^[^ ^105^ ^]^ and human fingernails,^[^ ^106^ ^]^ supporting the triboelectricity generation abilities of various keratin‐based BW materials. For example, keratin in wool and horn possesses intrinsic piezoelectric coefficients, d_14_, of ≈0.1 and 1.8 pC N^−1^, respectively.^[^ ^107^ ^]^

In animals, collagen is found in the skin, bone, cartilage, and tendons. The interlocked chain‐like structure of collagen with three polypeptide chains twisted around each other results in collagen fibers (Figure 7f) that can be arranged in various ways to develop different body tissues (e.g., a parallel arrangement in tendons and a randomly scattered arrangement in the skin). Similar to keratin, collagen is composed of polypeptide chains, resulting in similar FTIR spectra. The triboelectric behavior of collagen‐enriched chicken skin, a common BW material, has been investigated.^[^ ^108^ ^]^ FTIR analysis confirmed the presence of abundant collagen in chicken skin, exhibiting amide A, B, I, II, and III bands corresponding to various electron‐donating functional groups, such as C═O, C–N, and N–H (Figure 7g). Consequently, the corresponding TENG achieved high triboelectric voltages that varied with the frequency of mechanical vibration (Figure 7h). The structural stability of collagen was investigated in fish scales, fins, scales, bladders, and fish scale‐derived gelatin, a hydrolyzed form of collagen. The –CONH groups in the polypeptide chains of collagen fibers can produce piezoelectricity under an external strain. In mammals, the d_14_ values of skin and bone are ≈0.2 pC N^−1^, whereas that of muscle tendons is 2.0 pC N^−1^.^[^ ^107^ ^]^ In contrast, fish byproducts generate greater strain‐induced polarization with d_14_ values of 22, −3, and −5 pC N^−1^ for the bladder, skin, and scales, respectively.^[^ ^109^, ^110^ ^]^ Among BW materials, the eggshell membrane is the most widely used in mechanical energy harvesting because it exhibits the maximum d_14_ value of ≈23.7 pC N^−1^, originating from a combination of collagen fibers with other proteins.^[^ ^111^ ^]^

## 3 TENGs Based on BW Materials

### 3.1 Untreated and Treated BW‐TENGs

#### 3.1.1 Cellulose‐based BW‐TENGs

**Figure 8:**
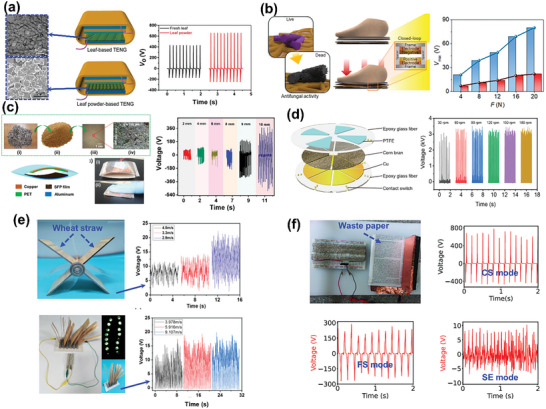
Structures and performance characteristic of cellulose‐based BW‐TENGs: a) leaf‐powder‐based TENG. Reproduced with permission.^[^ ^112^ ^]^ Copyright 2019, Elsevier; b) electrospun tree‐root bark‐based TENG. Reproduced with permission.^[^ ^115^ ^]^ Copyright 2022, Elsevier; c) sunflower‐husk‐based TENG. Reproduced with permission.^[^ ^118^ ^]^ Copyright 2021, Elsevier; d) corn‐bran‐based rotary TENG. Reproduced with permission.^[^ ^120^ ^]^ Copyright 2022, Elsevier; e) wheat‐straw‐based TENGs with windmill‐ and lawn‐like designs. Reproduced with permission.^[^ ^122^ ^]^ Copyright 2021, Elsevier; and f) wastepaper‐based TENG in CS, FS, and SE modes. Reproduced with permission.^[^ ^17^ ^]^ Copyright 2023, Wiley‐VCH.

Plant‐based cellulosic BW materials include forest/garden waste (e.g., wood, bark, dry leaves, and flowers), household waste (e.g., fruit and vegetable peels), agrowaste (e.g., husks and straws), and office wastepaper. In a comparative analysis, powdered leaves, which have a larger effective surface area, exhibited significantly better TENG performance than dry leaves (**Figure** **8**a).^[^ ^112^ ^]^ The higher voltage output of dry leaves can be attributed to the absence of water, which reduces electron generation in fresh leaves. Sundried cactus nopal powder applied to a Cu tape generated a triboelectric power of ≈556.72 µW m^−2^.^[^ ^113^ ^]^ Disadvantageously, the direct attachment of this powder to the tape increased the wear rate of the device. Following a similar approach, cleaned and dried *Ulva lactuca*, a coastal BW, was ground twice to obtain a fine powder, evenly distributed on the adhesive side of an Al substrate, and pressed multiple times using a roller to evenly cover the entire substrate and improve adhesion.^[^ ^114^ ^]^ Finally, an air blower was used to remove loose particles to avoid fallout during triboelectrification. The uniformly distributed BW particles generated a triboelectric voltage of ≈875 V, which was maintained for 8 days. The dispersion of a desiccated plant powder in a biocompatible polymeric medium can increase the durability of TENG devices. A biodegradable TENG layer was prepared using a PVA–flower extract composite.^[^ ^25^ ^]^ The resulting TENG, which continuously generated a steady output of ≈144.4 µW for 8 months, was successfully employed in a self‐powered step‐counter device. The powdered root bark of *Ulmus davidiana* var. japonica was mixed with a polycaprolactone solution and electrospun to prepare a nanofibrous mat with enhanced stability as a durable TENG layer (Figure 8b).^[^ ^115^ ^]^ The corresponding TENG generated a steady voltage of ≈69 V for up to 100 000 continuous tapping cycles. When integrated into an insole as a step‐counter and biomechanical energy harvester, this biocompatible TENG also protected the athlete's foot from fungus owing to its antifungal activity.

Agriculture has historical importance in civilization with an undisputable role in the current global economy. Nevertheless, increasing agroproduction to meet growing demands has resulted in adverse ecological consequences associated with waste production.^[^ ^116^ ^]^ Agrowaste accounts for a large proportion of solid waste globally, causing pollution and posing health risks when handled improperly.^[^ ^117^ ^]^ Agrowaste has a high content of cellulose, which has strong electron‐donating properties. A fine powder (≈25 µm) of BW sunflower husks was stuck to the adhesive side of Al tape to construct a BW‐TENG.^[^ ^118^ ^]^ Although high TENG outputs (488 V, 1200 µW, and 28.5 µA) were generated, the signal was unsteady owing to the nonuniform dispersion of the active material (Figure 8c). This nonuniform distribution likely results from the direct adherence of the powder to the tape. To avoid this issue, a fine powder of discarded rape straw was evenly dispersed in a PVA solution, and a biocompatible TENG layer was fabricated via drop casting.^[^ ^119^ ^]^ This BW‐TENG was successfully employed to harvest biomechanical energy from various human activities, generating a low but stable output of ≈78 V.

The incorporation of materials other than conventional tribo‐negative materials, such as PTFE and PI, can improve the energy‐harvesting characteristics while maintaining a stable output. The sheet‐like morphology of 2D graphitic carbon nitride (g‐C_3_N_4_) can increase the effective surface area between triboelectric layers, thereby promoting triboelectrification. A TENG was fabricated using waste coconut coir fibers dispersed in a PVA matrix as a tribo‐positive layer and spin‐coated g‐C_3_N_4_ as a tribo‐negative layer.^[^ ^19^ ^]^ Owing to enhanced CE, the TENG generated an output of ≈600 V and 1980 mW, which was effectively used as a self‐powered ultraviolet (UV) light sensor. In addition to materials, the design of TENGs can influence the energy‐harvesting performance. For example, rotary TENGs can improve energy harvesting from natural sources such as wind and flowing water. Agrowaste, such as corn husks, was utilized to prepare rotors for a rotary TENG, which was able to harvest a voltage of ≈3.2 kV upon rotation at 180 rpm under blowing air (Figure 8d).^[^ ^120^ ^]^ Similarly, discarded peanut shell powder was used in an air‐driven rotary TENG as a power source for an anticorrosion system.^[^ ^121^ ^]^ Moreover, a unique wheat‐straw‐based TENGs with windmill and bionic lawn‐like structures functioned as both a wind energy harvester and wind speed detector (Figure 8e).^[^ ^122^ ^]^ However, these TENGs could not harvest water‐flow energy because of the open structure and hydrophilicity of waste crop waste. As an alternative, a rotary TENG encapsulated in a cylindrical shell with a waterwheel‐like structure was prepared using cellulosic crop waste.^[^ ^77^ ^]^ This water‐driven TENG was capable of powering and controlling an automatic irrigation system.

Cellulose‐enriched paper and paper‐based products, which are discarded daily, can be utilized as triboelectric layers as well as flexible substrates for TENG preparation.^[^ ^123^ ^]^ Because paper is insulating, for use as a substrate, the paper surface is generally attached to a metal foil or painted with a metal ink.^[^ ^124^, ^125^ ^]^ Recently, a mixture of cellulose fibers extracted from discarded paper by immersion in water under mechanical stirring was used in a TENG, which generated a stable output voltage of ≈160 V in SE mode.^[^ ^126^ ^]^ Subsequently, this BW‐TENG was integrated with the pages of a book to harvest energy from simple actions such as page turning. As an active material, unprocessed paper typically offers poor TENG performance. To achieve enhanced tribo‐positive properties, a piece of commercial paper was treated with a PVDF solution. When used in a TENG as a tribo‐positive layer against a sheet of natural rubber as tribo‐negative layer, a voltage of ≈33 V was generated.^[^ ^127^ ^]^ This low output was attributed to a high content of α‐phase PVDF instead of the electroactive β‐phase. The inks used in paper printing generally contain polyester as adhesive, which is highly triboelectric.^[^ ^128^ ^]^ Thus, unprocessed waste journal or copy paper was directly attached to Cu tape to prepare TENGs.^[^ ^17^ ^]^ Owing to the ink and toner ingredients as well as the surface texture of the printed paper, these copy‐paper‐ and journal‐paper‐based TENGs exhibited excellent outputs of 774 and 432 V, respectively, in CS mode. In free‐standing (FS) and SE modes, the copy‐paper‐based TENG generated low voltages of ≈285 and 9 V, respectively, possibly because variations in the geometries of printed words and letters resulted in an irregular distribution of triboelectric effects (Figure 8f).

#### 3.1.2 Chitosan‐based BW‐TENGs

**Figure 9:**
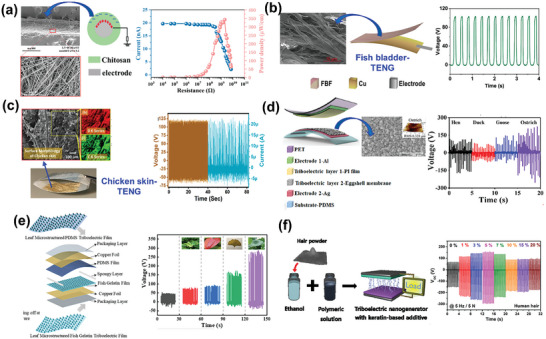
Structures and performance characteristics of collagen‐ and gelatin‐based BW‐TENGs: a) shrimp‐shell‐derived chitosan‐based TENG. Reproduced with permission.^[^ ^96^ ^]^ Copyright 2022, Springer Nature; b) fish‐bladder film‐based TENG. Reproduced with permission.^[^ ^137^ ^]^ Copyright 2020, American Chemical Society; c) chicken‐skin‐based TENG. Reproduced with permission.^[^ ^108^ ^]^ Copyright 2023, Springer Nature; d) eggshell‐membrane‐based TENG. Reproduced with permission.^[^ ^141^ ^]^ Copyright 2023, American Chemical Society; e) fish‐scale‐derived gelatin‐based TENG. Reproduced with permission^[^ ^144^ ^]^ Copyright 2023, Elsevier; and f) human‐hair‐based TENG. Reproduced with permission.^[^ ^146^ ^]^ Copyright 2022, American Chemical Society.

Biodegradable chitosan derived from the hard shells of marine creatures, a common seafood waste product, can be used as an electron‐donating element for TENGs.^[^ ^129^ ^]^ Chitosan exhibits excellent biocompatibility and antimicrobial properties, expanding its applicability into the biomedical field.^[^ ^130^ ^]^ The potential of a biocompatible and flexible chitosan‐based TENG for biomedical applications was investigated by placing the device directly on human skin.^[^ ^131^ ^]^ Toward the development of self‐powered baby‐care products, an edible TENG was prepared using chitosan‐ and cellulose‐based complexes,^[^ ^132^ ^]^ which had no effect on baby or human health upon consumption. Owing to its biocompatibility, a crab‐shell‐derived chitosan/PVA‐based composite TENG was used to monitor oral health.^[^ ^35^ ^]^ The TENG generated a low voltage (≈20 V), which was attributed to the presence of the polymeric medium, but the output was stable against a wide range of applied forces. Chitosan also offers good mechanical properties, which are advantageous for fabricating smart‐textile‐based electronics. Discarded shrimp shells were chemically refined to obtain pure chitosan, which was then electrospun with a conducting nylon yarn for use as a self‐powered sensing fiber.^[^ ^96^ ^]^ The soft fiber with a length of 20 cm was able to endure a weight of 200 g despite being extremely lightweight (0.023 g). The fiber was used in smart‐home applications with a triboelectric output of ≈342 µW cm^−1^ (**Figure** **9**a).

#### 3.1.3 Collagen‐ and Gelatin‐Based BW‐TENGs

Collagen‐enriched fish byproducts have high inherent piezoelectricity, resulting in outstanding performance as TENG layers. Without additives, demineralized fish scales with an intrinsic dipole orientation of 19% can generate a piezoelectric voltage of ≈14 V.^[^ ^133^ ^]^ When hybridized with PVDF, this BW material can nucleate the electroactive γ‐phase of PVDF via epitaxial crystallization as a synergistic effect.^[^ ^134^ ^]^ A single fish scale can be used as the tribo‐positive layer because of its electron‐donating nature. However, the choice of electronegative layer is crucial because the triboelectric output voltage depends on the electronegativity difference between the triboelectric layers. With a fish scale as the tribo‐positive layer, eggshell membranes, dog hair, or tree cotton as the tribo‐negative layer generated only ≈1 V owing to the small electronegativity difference.^[^ ^135^ ^]^ In contrast, pairing the fish scale with a PTFE tribo‐negative layer maximized CE, resulting in the generation of 50 V (peak‐to‐peak). Moreover, this device exhibited good thermal stability up to ≈200 °C. Similar studies on discarded fish fins revealed a relatively low triboelectric voltage of ≈8 V owing to decreased stress‐induced polarization.^[^ ^136^ ^]^ The bladder, which has the highest piezoelectricity among fish byproducts, can induce high polarization during triboelectrification. A fish‐bladder‐based TENG generated a voltage of ≈106 V with a transferred charge of 40 nC (Figure 9b).^[^ ^137^ ^]^ This TENG, which produced a maximum power of ≈0.39 mW with a dielectric constant of ≈23 at 1 kHz and remanent polarization of 0.069 C m^−2^, was subsequently fixed on a robotic arm for noncontact position‐detection applications.

Other types of food waste, such as chicken skin and eggshell membranes, also exhibit high tribo‐positivity. Dry chicken skin, with a surface roughness of ≈112.5 nm and an electronic potential of 480–568 mV, can generate a triboelectric signal of ≈123 V (Figure 9c).^[^ ^108^ ^]^ Eggshell membranes, which comprise a combination of collagen types X, V, I, and glycosaminoglycans, are extremely stable in alcoholic and aqueous media but degrade quickly at higher temperatures. Furthermore, these membranes have a maximum stress and strain of∼≈6.59 MPa and ≈6.98, respectively.^[^ ^138^ ^]^ A chicken eggshell membrane obtained from unboiled eggshells generated a triboelectric signal of∼≈100 V when tapped against a PTFE layer.^[^ ^139^ ^]^ The TENG performance of this material was enhanced when combined with ZnO, generating ≈250 V against a PTFE layer.^[^ ^140^ ^]^ Variations in the surface morphology of eggshell membranes greatly affect the triboelectric performance. Eggshell membranes from hens, ducks, geese, and ostriches exhibit differences in surface roughness, porosity, interspaces, and effective surface area.^[^ ^141^ ^]^ When used as a triboelectric layer, the ostrich eggshell membrane exhibited the highest surface roughness of ≈0.328 µm and electronic potential of 9.5 kV, thereby generating the highest triboelectric voltage of 280–300 V (Figure 9d). Further, this TENG exhibited high stability and durability under continuous vibration of 40 N at 3 Hz for over 9000 cycles. When attached to the human body, the TENG could harvest mechanical energy from various body movements.

Fish gelatin, a hydrolyzed version of collagen derived from fish byproducts, contains abundant electron‐donating functional groups but has a smooth surface that reduces friction during TENG operation. Thus, fish gelatin/PDMS films only generated a TENG voltage of ≈20 V.^[^ ^142^ ^]^ When the surfaces of both films were roughened by embedding PTFE powder, the TENG voltage increased significantly ≈125 V. As chemical treatment can achieve precise roughness between friction layers, this technique enabled the generation of a high TENG output voltage of 500 V, which could simultaneously light 300 light‐emitting diodes (LEDs) and was successfully utilized in HMIs.^[^ ^143^ ^]^ A pair of fish gelatin films, one reacted with trichloro(1*H*,1*H*,2*H*,2*H*‐tridecafluoro‐*n*‐octyl)silane (FOTS) and then treated under oxygen plasma and the other functionalized with dopamine, were employed as triboelectric layers to form a biocompatible TENG that naturally degraded in soil in ≈25 days. Despite improving the TENG output, these conventional surface treatment methods are expensive, complicated, and harmful to the environment. As an alternative, patterned fish gelatin films were prepared using a leaf micromold without any other treatment.^[^ ^144^ ^]^ Different natural leaves were used as molds to introduce various patterns onto films. The lotus‐leaf‐patterned bionic film produced a maximum voltage of ≈320 V, which was almost 5.8 times that of the nonpatterned film (Figure 9e).

#### 3.1.4 Keratin‐Based BW‐TENGs

Directly using discarded human hair as a triboelectric layer in a roller‐type TENG generated an output voltage of ≈23 V,^[^ ^145^ ^]^ whereas finely chopped dog hair adhered to Al foil produced a voltage of ≈200 V (peak‐to‐peak) in the CS mode.^[^ ^105^ ^]^ Furthermore, spin‐coated human hair pulp produced a triboelectric voltage of ≈103 V (peak‐to‐peak).^[^ ^104^ ^]^ When powdered fingernail was attached to a Cu foil, the resulting TENG produced a voltage of ≈53.2 V.^[^ ^106^ ^]^ These investigations indicate that crushing untreated BWs improves the triboelectric performance. Untreated‐keratin‐based BW cannot produce a high power density, stable triboelectric output, or durable devices owing to its unstable layering. A fine powder of discarded human hair prepared using a razor and a mortar and pestle was homogeneously dispersed in an ethanol/polyvinyl butyral (PVB) solution^[^ ^146^ ^]^ and then spin‐cast on an Al substrate. This triboelectric layer generated a high voltage (≈296 V) and current (≈37.6 µA) (Figure 9f). FTIR analysis supported the improved triboelectric performance, as amide I peaks corresponding to C═O stretching vibrations were significantly enhanced in the hair/PVB composite system. The BW‐TENG with a power density of 0.6 mW cm^−2^ remained stable after several weeks at protein denaturation temperatures of 40 and 60 °C.

### 3.2 Carbonized BW‐TENGs

**Figure 10:**
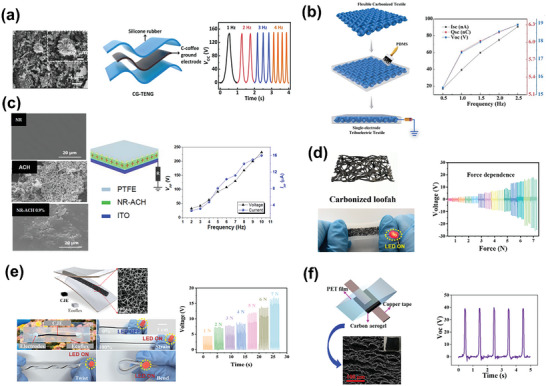
Energy‐harvesting performance of biocompatible TENGs prepared using a) carbonized waste coffee grounds. Reproduced with permission.^[^ ^147^ ^]^ Copyright 2021, Elsevier; b) carbonized cotton cloth. Reproduced with permission.^[^ ^149^ ^]^ Copyright 2020, Royal Society of Chemistry; c) carbonized waste human hair. Reproduced with permission.^[^ ^150^ ^]^ Copyright 2022, MDPI; d) carbonized loofah. Reproduced with permission.^[^ ^156^ ^]^ Copyright 2023, Elsevier; e) carbonized plant fiber. Reproduced with permission.^[^ ^157^ ^]^ Copyright 2023, Elsevier; and f) carbonized plant‐derived cellulose. Reproduced with permission.^[^ ^160^ ^]^ Copyright 2021, Elsevier.

Alkaline/acidic solutions can remove certain impurities (e.g., lignin), thereby providing a porous and pure template for carbonization. Alkali‐treated coffee waste, exhibiting a homogeneous porous morphology after sintering at high temperatures,^[^ ^147^ ^]^ was used to fabricate a TENG that produced a triboelectric voltage of ≈150 V (**Figure** **10**a). When worn on different body parts, this extremely stretchable TENG harvested biomechanical energy from various gestures, including stepping, patting, folding, and bending. When encapsulated in silicon rubber, the TENG exhibited outstanding stretchability, generating a current density of≈1.1 mA m^−2^ under an external strain of 150%. As an alternative to time‐consuming chemical treatments, plasma treatment offers fast and easy carbonization.^[^ ^148^ ^]^ A TENG active layer based on an air‐plasma‐treated cotton fabric decorated with graphene produced a triboelectric voltage, current, and charge of∼≈17.8 V, 83 nA, and 6.2 nC, respectively (Figure 10b).^[^ ^149^ ^]^

C materials can also be used as additional fillers in polymeric TENGs to enhance the triboelectrification performance. A natural‐rubber‐based TENG was modified with carbonized pretreated human hair discarded at a barber shop.^[^ ^150^ ^]^ The high conductivity arising from a suitable amount of sp^2^ C resulted in a hybrid TENG with outputs of∼≈232 V and 242 mW m^−2^, which are nearly three times those generated by natural rubber alone (Figure 10c). The harvested energy was sufficient for charging a 47 µF capacitor up to 3 V within 550 s.

Several biomass materials have intrinsically porous, 3D interconnected structures.^[^ ^151^ ^]^ The direct carbonization of such materials produces C materials that maintain these inherent structures, which are favorable for strain‐related and other flexible electronic applications.^[^ ^152^ ^]^ Because of its well‐arranged 3D network‐like microstructure, natural loofah is an excellent candidate for carbonization to produce materials with applicability in electromagnetic shielding, energy storage, piezoresistive tactile sensing, and TENGs.^[^ ^153^, ^154^, ^155^ ^]^ A sheet of carbonized loofah encapsulated in Ecoflex generated a fast and stable response against the minimum applied strain(∼≈0.01%), demonstrating its suitability for detecting weak physiological signals.^[^ ^156^ ^]^ With this hybrid material as a TENG active layer, a signal of ≈19.3 V was generated under a mechanical vibration of 7 N (Figure 10d).

Symmetrical fibers from the plant *Juncus effusus* were directly carbonized to maintain their 3D fractal arrangement, that is, a self‐similar interconnected structure with expanding symmetry.^[^ ^157^ ^]^ When this material was applied as a strain sensor with an Ecoflex coating, a high sensitivity was achieved, corresponding to a 1100% change in resistance at a strain of 80%. When applied as a TENG material in the CS mode, an electrical signal of ≈17.5 V was generated under an external force of 7 N (Figure 10e).

C aerogels synthesized using traditional methods are extremely fragile and dense, making them inappropriate for TENG operation.^[^ ^158^, ^159^ ^]^ These limitations were overcome by carbonizing dry‐plant‐derived cellulose, glucose, and dicyandiamide, as the prepared C aerogel had a density of 0.009 g cm^−3^.^[^ ^160^ ^]^ This aerogel exhibited good fatigue resistance and mechanical stability when subjected to strains of 10%–90%. In a TENG, the aerogel effectively generated triboelectric outputs of ≈38 V, 3 µA, and 12 nC (Figure 10f).

**Figure 11:**
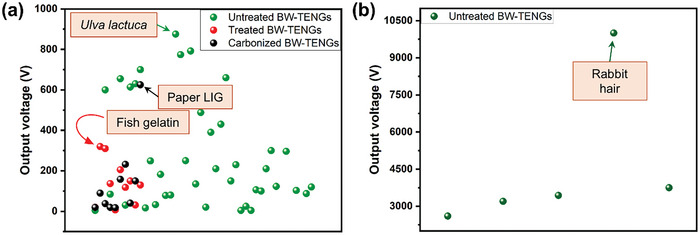
Comparison of the output voltages of untreated, treated, and carbonized BW‐TENGs: output voltages a) below 1 kV and b) above 1 kV.

The output performance of untreated, treated, and carbonized BW‐TENGs are summarized in **Tables** **2**, **3**, **4**, respectively. In addition, to facilitate comparisons, **Figure** **11**a,b depict the output voltages of these systems. Notably, all the BW‐TENGs exhibiting voltages of ≈kV were based on untreated BW materials and rotary TENG structures.

**Table 2:** Untreated BW‐TENGs and their output performances.

No.	BW material used	Forms used	Voltage (V)	Current (µA)/Current density	Power/Power density	Application	Ref.
1	*R. vesicarius* leaves	Leaves powder extract	3.86	3.78	0.1894 µW cm^−2^	Energy harvesting	[251]
2	Agricultural plant debris	Fiber	2600	90	1.24 W m^−2^	Plant growth monitoring, soil sensor, automatic irrigation system	[77]
3	Corn husk, Coconut coir	Pieces in µm size range + 2D‐gC_3_N_4_	600, ≈600	0.79 mA, 11.47 mA	32.75 mW cm^−2^ 495 mW cm^−2^	Photo‐detection	[19]
4	*Clitoria ternatea* flower	Flower extract + PVA	84.68 (peak‐to‐peak)	6.36	144 µW	Humidity sensing, touch‐sensitive counting	[25]
5	Corn husk	Husk powder + Methylecellulose	3200	–	–	Agro‐sensing	[120]
6	*Delonix Regia* flowers	Dry flower powder	655	59	220.2 µW cm^−2^	Powering 210 LEDs, capacitor charging	[24]
7	Rose petal	Single piece	30.6	0.78	27.2 mW m^−2^	Water drop energy harvesting	[23]
8	Almond shell, Walnut shell, Pistachio shell	Powder	614, 630, 700	85, 87, 95	341.33 µW cm^−2^ 385.33 µW cm^−2^ 416.14 µW cm^−2^	Humidity sensing	[29]
9	Dry nopal leaf	Powder	16.4	–	556.72 µW m^−2^	Powering 18 LEDs, capacitor charging	[113]
10	Peanut shell	Powder	248.9	39.4	365 mW m^−2^	Powering 228 LEDs, anti‐corrosion system	[121]
11	Paper	Single piece coated with PVDF	≈33	3	21 mW m^−2^	Energy harvesting	[127]
12	Leek skin	Half‐cell film from monolayer cell film	182	0.83 mA m^−2^	35 W m^−2^	Humidity sensing, acetone sensing	[18]
13	Rape straw	Powder + PVA	78	5.3	104.5 µW	Motion monitoring, Gait sensing	[119]
14	Root bark of *U. davidiana* var. *japonica*	extract/polycaprolactone electrospun	80	–	9 mW m^−2^	Antifungal shoe insole‐based energy harvester	[115]
15	*Ulva lactuca*	Dry powder	875	52	272.72 µW cm^−2^	Energy harvesting, powering 160 LEDs	[114]
16	Wasted printed paper	Single piece	774	3.92 mA	43.5 W m^−2^	Powering 300 LEDs, charging capacitor	[17]
17	Wheat straw	Straws layered	250	12	404 mW m^−2^	Wind energy harvesting in windmill, and bionic lawn structure	[122]
18	Used tea leaves	Milled	792	42.8	488.88 µW cm^−2^	Energy harvesting in honeycomb lantern structure, Powering 179 LEDs	[250]
19	Tomato peel	Single piece	135	81	3750 µW	Energy harvesting	[26]
20	Sunflower husk	Powder	488	28.5	1200 µW	Humidity sensing	[118]
21	Plant protein	Film	20.3	1.26	–	Plant growth promoting system	[206]
22	Peanut shell	Powder	390	14	1.3 mW	Powering 150 LEDs	[28]
23	Waste paper	Uniform fiber mixture	96–210	1‐8	171 mW m^−2^	Powering 36 LEDs	[126]
24	Leaves	Fresh leaf, Leaves powder	430, 660	15, 26	‐ 17.9 mW	Powering 868 LEDs	[112]
25	Deciduous Leaf	Dry single piece	150	4.2	72.2 µW	Energy harvesting	[22]
26	Hosta leaf	Fresh leaf	230	9.5	45 mW m^−2^	Energy harvesting	[252]
27	Used paper wipes	Wipe coated with carbon	–	3.5	0.61 mW	Morse code generator, self‐powered keyboard	[165]
28	Waste textiles	Fibers knitted together	4.2	2.7 nA	–	Sports judgement system	[178]
29	Fish scale	1 cm × 1 cm piece	≈25	1.7	–	Powering 90 LEDs	[135]
30	Fish fin	3 cm × 4 cm piece	≈4	1.1	–	Powering 65 LEDs	[136]
31	Fish bladder	Dry single piece	106	7.3	0.39 mW	Proximity sensing	[137]
32	Chicken eggshell membrane	Single piece	≈100	1.3	0.25 W m^−2^	Powering LEDs	[139]
33	Eggshell membrane	Single piece dipped in ZnO solution	≈210	1.2	–	–	[140]
34	Ostrich eggshell membrane	2 cm × 2 cm single piece	≈300	0.6 µA cm^−2^	270 µW cm^−2^	Body motion monitoring	[141]
35	Chicken skin	Single piece	123	20	0.2 mW cm^−2^	Physiological signal, temperature monitoring	[108]
36	Rabbit hair	Numerous hair strands stuck between rotor gaps	1200–3440	12	–	UV sterilization system	[32]
37	Human hair	Hair powder + polymeric matrix	296	37.6	0.6 mW cm^−2^	Capacitor charging, small device operating	[146]
38	Rabbit hair	Numerous hair strands stuck on rotor plates	≈10 000	≈40	1200 mW	Curtain purification system	[207]
39	Human hair	Pulp	103	10.9	60 mW m^−2^	Energy harvesting	[104]
40	Rabbit fur	Numerous hair strands stuck on rotor plates	–	15	11.9 mW	Wind energy harvesting, smart farming	[30]
41	Rabbit fur	Numerous hair strands stuck on rotor plates	3750	28	56.79 mW	Agro sensing, weather monitoring	[16]
42	Human finger nail	Powder	87.3	3.2	122 mW m^−2^	Energy harvesting	[106]
43	Snake ecdysis	Single piece	120	60	6250 µW	Energy harvesting	[103]

**Table 3:** Treated BW‐TENGs and their output performances.

No.	BW material used	Form	Voltage (V)	Current (µA)	Power/Power density	Application	Ref.
1	Crab shell	Chitosan + PVA	20	200 nA	0.45 µW	Oral health monitoring	[35]
2	Fish scales	Gelatin	320	0.80	57 µW cm^−2^	Motion monitoring	[144]
3	Fish scales	Modified gelatin	310	2.5	100 µW cm^−2^	Motion monitoring, game controller	[143]
4	Sugarcane bagasse	Cellulose nanofiber + Activated carbon + Natural rubber	137	12.1	2.74 W m^−2^	Powering 80 LEDs, capacitor charging	[37]
5	Shrimp shell	Chitosan fibers (electrospun)	6.4	17.4 nA	342 µW cm^−1^	Smart home control	[96]
6	Wasted paper	Cellulose nanocrystal + Methylecellulose (commercial)	205	18	1 W m^−2^	Real‐time monitoring	[41]
7	Tanned leather shavings	Hydrolyzed collagen + PVA + Ag nanowires	118	3.8 nA	21.06 mW m^−2^	Powering 30 LEDs	[46]
8	Wood pulp	Nanofibrillated cellulose	150	0.74–2.80	0.33 mW	Touching password switch	[162]
9	Wood powder	Citric acid‐crosslinked cellulose‐lignin mixture	31	0.2	10 mW m^−2^	Smart ward and medical monitoring	[181]
10	Fish scales	Gelatin film	130	0.35	45.8 µW cm^−2^	Physiological signals monitoring	[142]

**Table 4:** Carbonized BW‐TENGs and their output performances.

No.	BW material used	Form	Voltage (V)	Current (µA)/Current density	Power/Power density	Application	Ref.
1	Loofah	Direct carbonized	19.3	0.22	0.21 µW cm^−1^	Strain sensing, physiological signal monitoring	[156]
2	Human hair	Activated carbon + Natural rubber	89.6	6.9	242 mW m^−2^	Powering 44 LEDs, capacitor charging	[150]
3	Biomass	N‐doped carbon aerogel	38	3		Strain sensing, physiological signal monitoring	[160]
4	*Juncus effusus* fiber	Direct carbonized	19	480 nA	1.3 µW	Strain sensing, physiological signal monitoring	[157]
5	Cellulose (cotton) fabric	Carbonized + RGO	17.8	83 nA	0.8 µW cm^−1^	Pressure sensing, pulse and motion monitoring	[149]
6	Phytic acid from rice bran, saccharose from sugarcane	Phosphorus‐doped mesoporous carbon	158	2.26 µA cm^−2^		Sitting posture monitoring	[167]
7	Lignin fiber	Carbon fiber	232	17 mA m^−2^	1.6 W m^−2^	Impact sensing of packages	[60]
8	Paddy straw	Graphene Quantum Dots + ZnO	40	2 µA cm^−2^	–	UV‐light sensing	[62]
9	Coffee waste	Carbon	150	2.1	63.8 mW m^−2^	Real‐time monitoring	[147]
10	Paper	LIG (electrode)	625	20 mA m^2^	2.25 mW m^−2^	Energy harvesting	[72]

## 4 Emerging Bioelectronics Based on BW‐TENGs

### 4.1 HMIs

**Figure 12:**
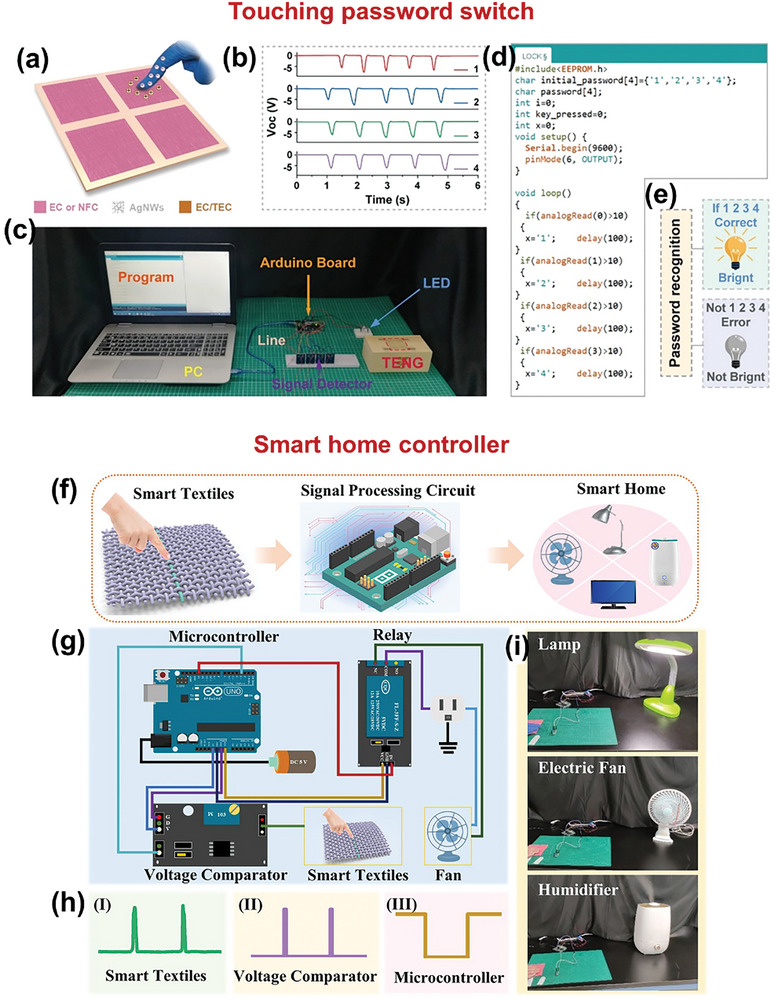
Cellulose‐based TENG for tactile password recognition: a) four TENGs used to prepare the password input system; a positive signal was generated by each TENG when touched with a finger wearing nitrile rubber glove, b) *V*_oc_ generated by each TENG when touched 5 times with a finger wearing nitrile rubber glove, c) digital photograph of the password recognition switch system, d) program for setting the password, which was decrypted by touching the TENGs in the sequence “1234,” and e) detection of password recognition via lighting of an LED. Reproduced with permission.^[^ ^162^ ^]^ Copyright 2022, Elsevier. Smart‐home controller: f) schematic of the BW‐TENG‐based smart home controller switch, g) schematic of the entire device, h) voltages generated by the I) TENG and II) comparator, and III) signal transmitted to the relay by the microcontroller, and i) digital photographs of appliance control using the TENG. Reproduced with permission.^[^ ^96^ ^]^ Copyright 2022, Springer Nature.

The sensitivity of HMIs can be exploited to develop smart appliances for intelligent home management.^[^ ^161^ ^]^ A touch password switch system was developed using a biocompatible TENG fabricated with wood‐pulp‐derived cellulose via the TEMPO oxidation method.^[^ ^162^ ^]^ Four SE TENGs were mounted on a transparent multilayer cellulose film to prepare the smart password input system (**Figure** **12**a). Using a finger wearing a nitrile glove, each TENG was touched to observe the generation of stable voltages (Figure 12b). The TENGs were assigned numbers (1, 2, 3, and 4) before being sequentially integrated with other components (Figure 12c). Subsequently, a password lock/unlock pattern was programmed on a computer with “1234” as the initial password (Figure 12d). When the TENGs were touched in a preset sequence, the password protection was unlocked, and the LED lit up (Figure 12e).

Electrospun fibers were prepared using refined chitosan derived from prawn shells mixed with dichloromethane and trifluoroacetic acid.^[^ ^96^ ^]^ The TENG fabricated by wrapped the prepared fiber around Ag‐plated nylon yarn was used to control home appliances, as shown schematically in Figure 12f, following the circuit in Figure 12g. The voltage signals generated by the smart textile (Figure 12h(i)) were converted into square‐wave signals (Figure 12h(ii)) by the comparator and then assessed by the microcontroller unit (MCU). Depending on the difference between the output signals, low/high commands were transmitted by the MCU to the switch of the corresponding appliance (Figure 12h(iii)). These commands were utilized as a self‐powered control system to turn ON/OFF home appliances, including a lamp, fan, and humidifier (Figure 12i).

**Figure 13:**
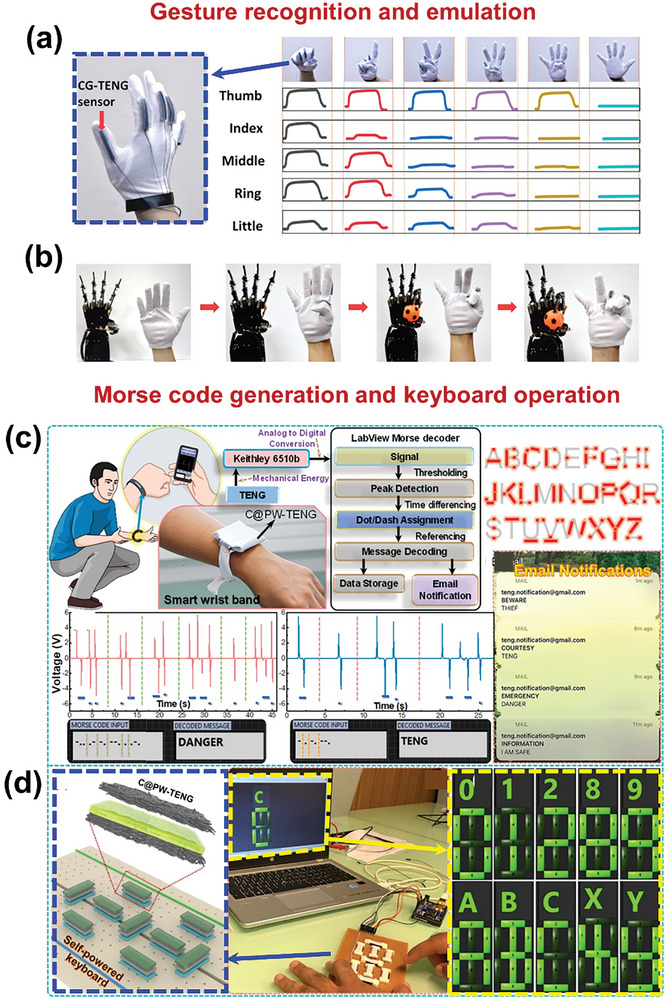
a) Electrical output of five carbonized ground coffee (CG)‐based TENG sensors corresponding to finger gestures representing the numbers 0, 1, 2, 3, 4, and 5, and b) human hand gesture emulation by a robotic hand. Reproduced with permission.^[^ ^147^ ^]^ Copyright 2021, Elsevier. c) Illustration of a carbon‐coated paper‐wipe‐based TENG in the form of a wearable smart wrist band for Morse code generation in emergency situations, and d) letter and number generation by a 9‐segment self‐powered keyboard prepared using the carbon‐coated paper‐wipe‐based TENG. Reproduced with permission.^[^ ^96^ ^]^ Copyright 2022, Springer Nature.

Carbonized waste coffee grounds were used to develop the electrode of a stretchable ecofriendly TENG for multiple HMIs.^[^ ^147^ ^]^ First, this device was applied as a pressure sensor that could rapidly detected a pressure of 1 kPa. Subsequently, it was employed as a computer game controller and smart vending coaster. Finally, the device was used to rapidly assess subtle gestures, motions, and physiological signals. Five TENG sensors were attached to human fingers to monitor transient gesture signals corresponding to numbers 0, 1, 2, 3, 4, and 5 (**Figure** **13**a). These signals were successfully used to command a robotic hand and imitate the corresponding gestures (Figure 13b).

The generation and transmission of Morse code is of global interest, mainly in emergency situations, owing to its security for telecommunications and information transmission. In this communication mode, distinct arrangements of dots and dashes, which correspond to the letters of the alphabet, are conveyed using electrical pulses or light signals. A waterproof TENG was first used for Morse code generation and transmission in 2019.^[^ ^163^ ^]^ The voltage signals were effectively visualized and decoded using an oscilloscope. In 2021, a TENG based on a waste laboratory coat was found to produce an output voltage, current, and power density of ≈31 V, 3.1 µA, and 11.9 mW m^−2^, respectively.^[^ ^164^ ^]^ This TENG was used as a fingerprint generator. Specifically, when touched with a finger, the device produced an output signal of ≈6 V, which was sufficient to power a liquid crystal display. In addition, a wireless Morse code generator was successfully devised using the TENG. A threshold of 2 µA was considered for Morse code generation (i.e., signals of <2 and >2 µA were perceived as dots and dashes, respectively). Recently, recycled paper wipes were used to prepare a cost‐effective and flexible BW‐TENG in the form of a smart wristband for the autonomous generation of Morse code.^[^ ^165^ ^]^ Paper wipes collected from a trash can were cleaned and brushed with a slurry of conducting C black in PVDF. This hybrid structure was used as a tribo‐positive layer along with two electrodes, whereas a waste PTFE coffee cup was used as the tribo‐negative layer. A LabVIEW model was applied to decode the Morse code dots and dashes pattern generated by the TENG. Subsequently, the messages were programmed to be sent via email (Figure 13c). Furthermore, nine TENGs were integrated into a nine‐section smart keypad to generate numbers and letters on a computer screen (Figure 13d).

### 4.2 Intelligent Monitoring

**Figure 14:**
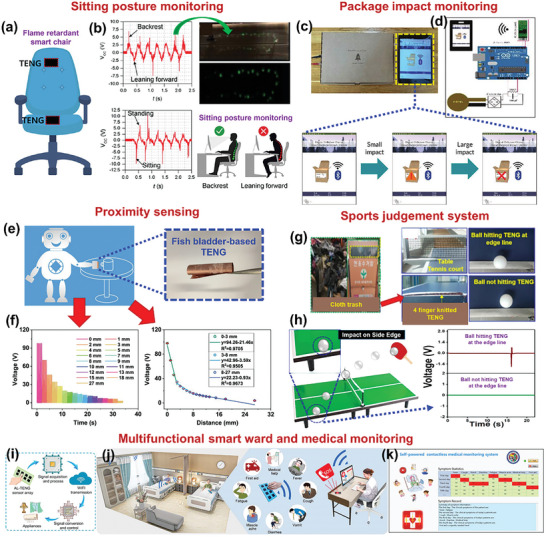
a) BW‐derived flame‐retardant TENG adhered to a chair for sitting posture monitoring, and b) voltage signal generated by the TENG at different sitting postures represented by LED lightening. Reproduced with permission.^[^ ^167^ ^]^ Copyright 2022, American Chemical Society. c) Real‐time images of a BW‐TENG applied in self‐powered remote impact‐monitoring system, with d) its device schematics. Reproduced with permission.^[^ ^60^ ^]^ Copyright 2022, Wiley‐VCH. e) Fish‐bladder‐based TENG for proximity sensing, and f) response curves of the TENG corresponding to contact and noncontact motions at different starting points (0–27 mm). Reproduced with permission.^[^ ^137^ ^]^ Copyright 2020, American Chemical Society. g,h) Sports judgment analysis using TENG prepared from discarded fabric. Reproduced with permission.^[^ ^178^ ^]^ Copyright 2022, Elsevier. i) Schematic diagram of the self‐powered smart ward system using lignocellulosic TENG, j) schematic representation of the self‐powered smart ward, and k) operating interface of the self‐powered contactless medical monitoring system Reproduced with permission.^[^ ^181^ ^]^ Copyright 2023, Royal Society of Chemistry.

TENGs are widely utilized for wireless intelligent monitoring. Touch‐sensitive TENGs can be integrated into seat cushions or chairs for the smart monitoring of sitting postures at different locations for the safety of disabled people or children. When attached to car seats, TENGs can be used for traffic safety observations.^[^ ^166^ ^]^ P‐doped mesoporous C was prepared hydrothermally using phytic acid and saccharose extracted from rice bran (chaff) and sugarcane in the KIT‐6 template.^[^ ^167^ ^]^ Subsequently, this material was combined with polyurethane foam to obtain a fire‐resistant TENG. When mounted on the seat plate and backrest of a chair (**Figure** **14**a), the TENG monitored and corrected sitting posture by lighting LEDs (Figure 14b).

As self‐powered impact sensors, TENGs can be applied for structural health monitoring. The effects of disasters, such as accidents, earthquakes, and tsunamis, can be minimized by detecting vibrations during impact. Various TENGs have been used as active impact sensors by analyzing the output corresponding to the impact of a free‐falling object.^[^ ^168^, ^169^ ^]^ When integrated into a smart package system (Figure 14c),^[^ ^60^ ^]^ a bio‐based TENG fabricated using a carbonized lignin fiber mat generated an output voltage and power of 232 V and 1.6 W m^−2^, respectively. The circuit system of this unit is shown schematically in Figure 14d. Using electronic modules, the system detected the impact exerted on the package during loading/unloading and transmitted real‐time information to a mobile app, allowing for the detection of package damage (Figure 14c).

Proximity or noncontact monitoring is among the greatest HMI breakthroughs for TENG applications in interactive somatosensory and robotics technology. This phenomenon was first demonstrated in 2018 using a PTFE‐based TENG in FS mode, which responded to the movement of a human hand at distances of 1–11 cm with a sensitivity of ≈315 V m^−1^.^[^ ^170^ ^]^ Other systems that have been investigated for noncontact gesture detection include inorganic/organic complexes, such as chemically etched graphene/indium tin oxide/PET,^[^ ^171^ ^]^ sputtered electron cyclotron resonance, oxygen plasma, through‐filter ion‐etched PET/electron‐induced perpendicular graphene patterned layers,^[^ ^172^ ^]^ and PVDF/MXene matrices.^[^ ^173^ ^]^ Fish bladder, which is typically considered food waste, is enriched with natural collagen fibers with a triple helical structure.^[^ ^174^ ^]^ With Cu nanoparticles deposited on one side, a fish bladder film was used to prepare a biocompatible flexible TENG in SE mode (Figure 14e). When employed as a noncontact proximity sensor, the TENG could detect a voltage signal of ≈3.8 V at a maximum vertical distance of 27 mm (Figure 14f).

During athletic training and competition, real‐time assessments of the players’ physical movements are necessary to monitor their performance and physical condition. Typical monitoring methods are based on optical, capacitive, resistive, thermal, chemical, and magnetic characteristics, each of which have limitations including a lack of flexibility, high rigidity, delayed assessment, low stability, bulk structures, and high costs.^[^ ^175^ ^]^ TENG‐based sports monitoring systems can overcome most of these issues with the additional advantage of not requiring an external power supply.^[^ ^176^, ^177^ ^]^ After chemically extracting gelatin from fish scale waste, a biodegradable TENG was fabricated by hydroxylating the gelatin film surface using oxygen plasma.^[^ ^143^ ^]^ The TENG was used for contact‐based motion monitoring during various body movements, including walking, running, and jumping. Additionally, the attachment of this TENG to a boxing glove allowed assessment of the boxer's movements and the force exerted during punching. Thus, the biodegradable transparent TENG has potential for smart sports management. A knitted TENG was prepared using fibers extracted from discarded fabrics.^[^ ^178^ ^]^ The TENG was utilized for the battery‐free recognition and differentiation of the impact produced by five boxers. This system was also used during a table tennis match for line review or edge judgment. When the TENG was fixed along the entire edge of the table tennis court (Figure 14g), an electrical signal was generated whenever the ball touched the edge (Figure 14h).

Owing to their multifunctionality, TENGs reduce the need for multiple units, thereby enhancing portability.^[^ ^179^, ^180^ ^]^ A multifunctional system was prepared using a biocompatible TENG as a remote control to operate electronic appliances and a tactile sensor to monitor the real‐time physiological condition of a person (Figure 14i,j). To fabricate the biodegradable SE‐mode TENG, a lignocellulosic slurry of powdered wood was used as the triboelectric active layer, and a mixture of lignocellulose and C powder was used as the electrode.^[^ ^181^ ^]^ The universality of this fabrication method was confirmed by preparing TENGs using straw from different plants (e.g., rice, corn, and wheat). Several 2 × 2 cm^2^ TENGs were combined in a control pad to develop a smart ward facility. Each patient could control the air conditioner, curtain, and lamp without assistance by touching the corresponding TENG on the control pad. Simultaneously, the patient's basic medical could be tracked and assessed without contact using TENGs (Figure 14k).

### 4.3 Healthcare Monitoring

**Figure 15:**
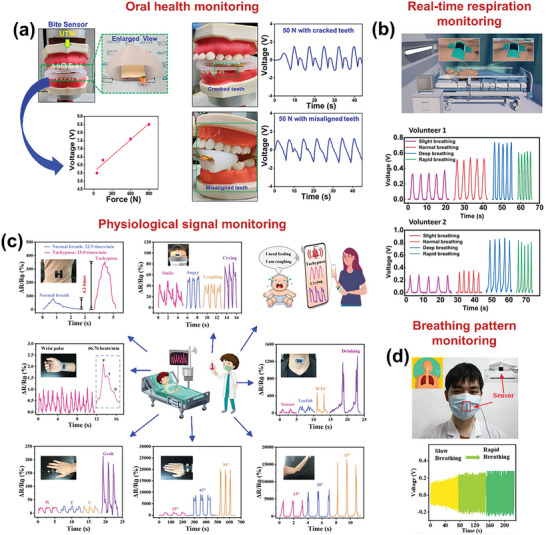
a) Real‐time oral health monitoring using crab shell derived chitosan based TENG refined food waste. Reproduced with permission.^[^ ^35^ ^]^ Copyright 2023, American Chemical Society. b) Real‐time respiration monitoring with TENG attached to abdomen of two patients. Reproduced with permission.^[^ ^41^ ^]^ Copyright 2022, Elsevier. c) Human motion monitoring via self‐powered strain sensing by carbonized loofah‐TENG sensor when attached on different parts of human body. Reproduced with permission.^[^ ^156^ ^]^ Copyright 2023, Elsevier. d) Photograph of the Cotton fabric‐TENG sensor after attaching inside a face mask and voltage response to breath for real‐time analysis. Reproduced with permission.^[^ ^187^ ^]^ Copyright 2023, Springer Nature.

Wearable oral health detectors have been designed to monitor dental issues during the early stages to prevent complications.^[^ ^182^ ^]^ These devices must be biosafe and toxin‐free because their constituents can enter the saliva and travel through the body.^[^ ^183^ ^]^ Waste crab‐shell‐derived chitosan was combined with PVA and integrated into a biocompatible BW‐TENG for oral health monitoring as shown in (**Figure** **15**a).^[^ ^35^ ^]^ The TENG was embedded in Ecoflex to protect it from damage caused by biting. The output of the device increased from ≈2 to ≈5 V when the bite force increased from 50 to 900 N. The output electrical signal of the TENG bite sensor exhibited distinct variations with healthy, cracked, and misaligned teeth, suggesting that this device is suitable for commercial applications.

Heart rate and pulse waves are crucial biological factors that can act as indicators for various physical conditions, such as cardiovascular disease, infection, and medication effects. Furthermore, variations in heart rate and pulse waves under different physical conditions can be used to identify stress‐related health conditions.^[^ ^184^ ^]^ However, the typical medical units for monitoring heart and pulse rates lack accessibility for regular checkups. Advantageously, by converting minute mechanical vibrations into electrical signals, TENGs can be employed for the self‐powered real‐time monitoring of these physiological signals. Moreover, as lightweight and flexible devices, TENGS are suitable as wearable health monitoring units.^[^ ^185^ ^]^ TENGs attached to the abdomen can track breathing patterns based on the expansion/contraction of the diaphragm. The difference between normal breathing and tachypnea can be clearly distinguished using TENG‐generated electrical signals.^[^ ^186^ ^]^ CNCs extracted from discarded printing paper via acid hydrolysis were used as a filler in a water‐soluble BW‐TENG.^[^ ^41^ ^]^ This biodegradable and cost‐effective TENG was employed as a disposable bandage sensor that continuously monitored physiological signals and respiration patterns (Figure 15b). Using the as‐prepared TENG, the respiration patterns of two volunteers under different conditions (e.g., deep and slow breathing) could be discriminated.

As TENGs produce different electrical signals when attached to different body parts, these devices can be employed for physiological signal monitoring. In particular, carbonized BW materials are considered to have potential for engineering biocompatible and sustainable electronic devices. These materials retain their original structures after heat treatment, which imparts excellent stretchability and good electrical properties.^[^ ^149^ ^]^ A *Juncus effusus* fiber was carbonized in an Ar environment and encapsulated in Ecoflex for application in both TENGs and physiological sensors,^[^ ^157^ ^]^ achieving a maximum voltage of ≈19 V and power of 1.3 µW. When attached to the human body, this TENG detected multiple body movements and the pulse. Waste coffee grounds, which were activated using a KOH solution and carbonized at 700 °C in a N_2_ environment, were used to prepare the conducting electrode of an eco‐friendly TENG with silicone rubber as the dielectric layer.^[^ ^147^ ^]^ The TENG harvested biomechanical energy to operate a calculator, an electronic watch, charge capacitors, and more than 40 LEDs. Moreover, this TENG could detect and differentiate numerous physiological signals, such as respiration, under various conditions. When worn on the wrist, the TENG detected three pulse‐wave peaks (P, T, and D). Using a similar method, dry loofah sheets were carbonized via a three‐stage process to obtain a 3D network‐like layer,^[^ ^156^ ^]^ which was then used to prepare a flexible TENG sensor. This sensor collected energy from various body movements and detected real‐time variations in physiological signals, such as normal breathing versus tachypnea, facial expressions, pulse rate, vocal information, and finger, wrist, and elbow movements (Figure 15c). When attached to a coat, the TENG was able to light up 50 LEDs through simple hand tapping.

A TENG was proposed for use as an e‐skin device in electrocardiography and healthcare monitoring. Lycra fabric sourced from clothing waste was immersed in a polydopamine solution for the in situ synthesis of polypyrrole.^[^ ^187^ ^]^ Subsequently, Ag NWs were sprayed onto the polypyrrole‐coated fabric and a humidity‐resistant TENG was fabricated. After washing 50 times, the output voltage of the TENG exhibited a minimal change, decreasing in from 0.48 to 0.32 V. The TENG attached to the body worked for efficient monitoring of human motion and breathing patterns (Figure 15d). Integration with an MCU allowed wireless real‐time step counting on a computer screen.

### 4.4 Humidity Sensing

**Figure 16:**
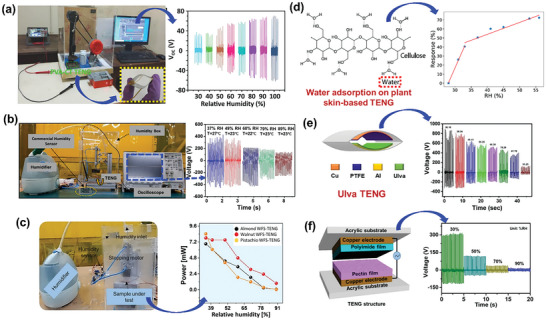
Humidity‐sensing properties of TENGs fabricated using a) *Clitoria ternatea* flower powder. Reproduced with permission.^[^ ^25^ ^]^ Copyright 2023, Elsevier. b) Sunflower husk. Reproduced with permission.^[^ ^118^ ^]^ Copyright 2021, Elsevier. c) Dry fruit shells. Reproduced with permission.^[^ ^29^ ^]^ Copyright 2022, Elsevier. d) Leek skin. Reproduced with permission.^[^ ^18^ ^]^ Copyright 2022, Elsevier. e) *Ulva lactuca*. Reproduced with permission.^[^ ^114^ ^]^ Copyright 2024, Elsevier. f) Citrus peel pectin. Reproduced with permission.^[^ ^192^ ^]^ Copyright 2022, Royal Society of Chemistry.

Recent advances involving triboelectrification under different humidity conditions have demonstrated the potential of TENGs as self‐powered humidity sensors. Owing to their chemical properties, some humidity‐active materials exhibit increased electrical output.^[^ ^188^, ^189^ ^]^ *CT* flower extract, which contains triterpenoids, anthocyanins, steroids, and flavonol glycosides with small quantities of metals (e.g., Na, Mg, Al, and Si), is an excellent candidate for advanced research applications. When this extract is paired with PVA, an artificial biodegradable polymer, synergistic effects provided enhanced physical properties that are beneficial for TENG applications.^[^ ^25^ ^]^ For example, the tribo‐positivity of the composite film was improved in the presence of humidity. Furthermore, hydrogen bond formation between the composite and H_2_O molecules increased the TENG output voltage from ≈80 to∼≈133.4 V (peak‐to‐peak) as the relative humidity (RH) increased from 30% to 100% in an enclosed chamber (**Figure** **16**a).

Fruit and vegetable peels, which are among the most commonly discarded items, are enriched with cellulose, which has excellent triboelectrification properties. Owing to high commercial demand, sunflower seeds are harvested in large quantities globally. However, sunflower seed processing produced a residue of 45%–50%, known as sunflower husks.^[^ ^190^ ^]^ The BW material was recycled as a powder stuck directly on Al tape to develop a BW‐TENG.^[^ ^118^ ^]^ The TENG output varied slightly at low and moderate humidities but decreased sharply at 60% RH (Figure 16b). Dry fruit shells (e.g., nuts) have high contents of O_2_‐rich lignin, cellulose, and carbohydrates,^[^ ^191^ ^]^ all of which can contribute to triboelectric charge generation. The finely powdered shells were deposited on an Al film to form a biocompatible TENG that generated a voltage of >600 V.^[^ ^29^ ^]^ Humidity significantly influenced the output power of this TENG (Figure 16c), likely because electron transfer between the TENG layers was blocked by adsorbed moisture.

A unique TENG‐based humidity sensor was fabricated by removing the skin of *Allium* plants (onions, scallions, and leeks) and halving the film of a single‐layer cell.^[^ ^18^ ^]^ The functional groups in the half‐cell film actively participated in humidity adsorption, as shown in Figure 16d. *Ulva lactuca*, a biomass that grows rapidly in coastal areas, has detrimental environmental effects, emitting a foul smell, causing underwater oxygen depletion, and harming sea creatures. This BW was recycled as an excellent triboelectric layer with an output power of ≈6750 µW.^[^ ^114^ ^]^ Moreover, the BW‐TENG output exhibited a sharp linear decrease with increasing humidity (Figure 16e).

In addition to numerous amino acids, tomato peels contain lutein and zeaxanthin, which have both piezoelectric and tribo‐positive characteristics. Waste tomato peels were directly attached to an Al foil strip to explore their TENG, piezoelectric nanogenerator (PENG), and TENG + PENG (TPENG) performance.^[^ ^26^ ^]^ The TPENG performance was unaffected up to 60% RH. At higher moisture contents, increased H_2_O absorption on the tomato peel surface restricted surface electrons, thereby reducing triboelectrification and the output voltage. This behavior is beneficial in two ways. First, the device can operate under harsh weather conditions at moderate humidities, and second, a high moisture content in air can be detected based on a decrease in the triboelectric output. Pectin is a nontoxic and tribo‐positive polysaccharide found in fruit peel. An aqueous mixture of citrus peel‐derived pectin powder and glycerol was used to prepare a micropatterned flexible tribo‐positive film.^[^ ^192^ ^]^ The output voltage and current of this BW‐TENG decreased rapidly with increasing RH (Figure 16f).

### 4.5 Photosensing

TENGs can be integrated with photodetectors to develop low‐cost, portable, and environmentally friendly self‐powered photosensors. Such sensors exhibit increased output signals under irradiation owing to the combined influence of the photoelectric effect and triboelectrification. Specifically, the photoelectric effect causes numerous electron–hole pairs to be generated in the photoactive layer. These newly generated charge carriers can participate in triboelectrification, thereby increasing the TENG output.^[^ ^193^ ^]^

**Figure 17:**
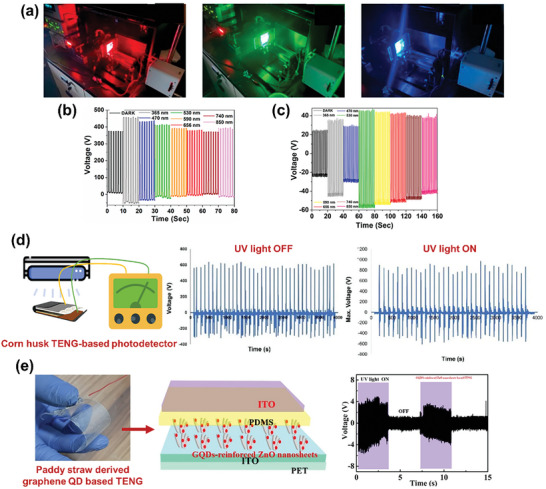
a) TENG‐based photo‐sensing lab set up under illumination of different wavelengths. Photo‐sensing properties of TENGs fabricated using b,c) human hair with different photosensitive materials. Reproduced with permission.^[^ ^195^, ^196^ ^]^ Copyright 2022 and 2023, MDPI and Elsevier. d) Corn husk. Reproduced with permission.^[^ ^19^ ^]^ Copyright 2023, Springer Nature. e) Graphitized paddy straw. Reproduced with permission.^[^ ^62^ ^]^ Copyright 2023, American Chemical Society.

Discarded human hair, which slowly degrades and often blocks sewage systems, is an excellent source of tribo‐positivity.^[^ ^194^ ^]^ Thoroughly washed and dried human hair was spin‐coated as a tribo‐positive layer in a self‐powered active photodetector.^[^ ^195^ ^]^ An α‐Fe_2_O_3_/PDMS composite layer was used as the tribo‐negative layer with α‐Fe_2_O_3_ as the photosensitive element. As shown in **Figure** **17**a, the TENG output varied significantly depending on the irradiation wavelength and intensity, confirming the good photodetection ability of the TENG (Figure 17b). This device exhibited a responsivity of 1.42 V mW^−1^ and a response time of less than 150 ms. The changes in the output signal agreed well with the UV‐vis absorption spectrum of the sensing element, and the highest response was achieved under irradiation with UV light at ≈365 nm. The same experiments were repeated with human hair as the tribo‐positive layer and mixed‐phase (rutile + anatase) black TiO_2_ as the photosensitive material, which has a remarkable charge‐trapping capability.^[^ ^196^ ^]^ Owing to the presence of oxygen vacancies and Ti^3+^ trapping centers, this broadband self‐powered photosensor could detect optical radiation from the UV to IR range with a responsivity of 606.8 V W^−1^ and the highest response in the visible spectrum (≈530 nm) (Figure 17c).

Coconut coir and corn husk contain large amounts of cellulose and lignin. In addition to retaining good strength and flexibility, these fibers are inherently tribo‐positive. BW‐TENGs were prepared with coconut coir or corn husk as the tribo‐positive layer^[^ ^19^ ^]^ and g‐C_3_N_4_ nanosheets as the tribo‐negative layer and photosensitive film. The coconut‐coir‐based TENG powered 55 commercial LEDs and generated higher output signals than the corn‐husk‐based TENG. Notably, the output of the corn‐husk‐based TENG increased by a factor of 1.5 under UV illumination (Figure 17d). Similar behavior was observed for the coconut‐coir‐based TENG, confirming the successful fabrication of a self‐powered UV photodetector.

Agrowaste can be carbonized to obtain highly pure materials for energy and sensing applications.^[^ ^197^ ^]^ Graphene quantum dots, synthesized from paddy straw BW via a hydrothermal process,^[^ ^62^ ^]^ were added to PDMS and ZnO nanosheets to fabricate a flexible BW‐TENG as a self‐powered UV light sensor (Figure 17e). The UV sensitivity of this device was confirmed by the wide optical bandgap of ZnO (≈3.37 eV) and the emission of blue light from the graphene quantum dots.

### 4.6 Agrosensing

The adoption of smart technologies for agriculture has encouraged precise and intelligent cultivation management via the monitoring of planting conditions and growth.^[^ ^198^ ^]^ To achieve practical, sustainable, and lucrative agricultural practices, TENGs are being incorporated into agricultural devices, where they can harvest mechanical energy from the surrounding environment (e.g., flowing water and wind).^[^ ^199^, ^200^ ^]^ Discarded lignocellulosic plant fibers were recycled as the triboelectric layer in a BW‐TENG to harvest water‐flow energy in farm fields.^[^ ^77^ ^]^ The BW‐TENG was integrated with a plant monitoring sensor and Bluetooth transmitter for the real‐time wireless transmission of plant growth, humidity, and soil moisture data. The device was connected to a water pump controller to achieve an automated irrigation system that could promote crop production by turning the water pump on/off automatically upon detecting the soil moisture content.

**Figure 18:**
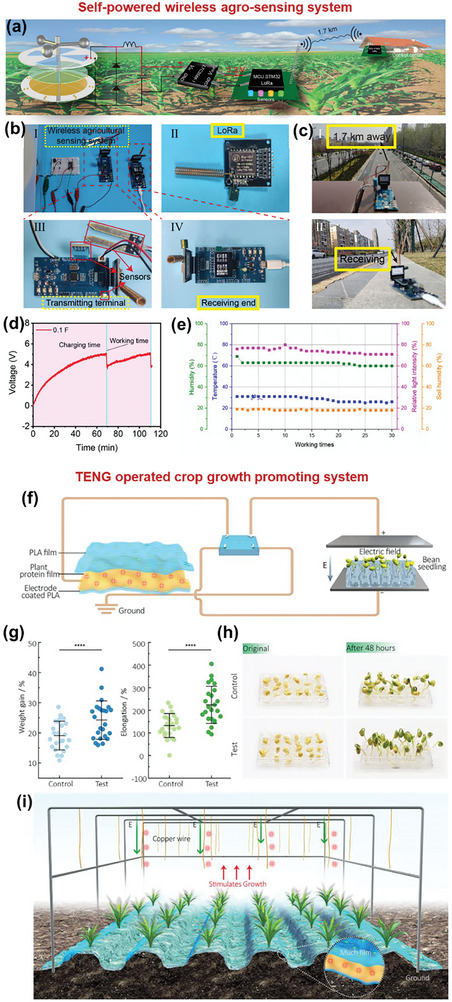
a) Schematic of corn husk‐TENG‐operated wireless agro‐sensing circuit system. b) Digital photo of each parts of the circuit system. c) Digital photo of 1.7 km wireless transmission of the sensing data. d) Charging curves of a 0.1 F supercapacitor charged by the TENG while driving the wireless agro‐sensing system. e) Photo of the sensing data shown on the host computer. Reproduced with permission.^[^ ^120^ ^]^ Copyright 2022, Elsevier. f) Schematic of the construction of a plant growth‐promoting system that generates space electric fields via plant protein‐based TENG. g) Percentage of weight gain (left) and elongation (right) in control (without electric field) and test (with electric field) groups after 48 h of growth. Whiskers indicate mean ± standard deviation for *n* = 24 bean seeds per group. Two‐sample *t*‐test; **** *p* < 0.0001. h) Typical photographs of beans with (test) and without (control) electric field application before and after 48 h of growth. i) Schematic of the TENG used as biodegradable mulch film to construct growth‐promoting system that generates space electric field for agriculture. Reproduced with permission.^[^ ^206^ ^]^ Copyright 2022, Elsevier.

Tribo‐positive lignocellulosic cornhusks possess good wear resistance and hydrophobicity. Pulverized cornhusks were mixed with methylcellulose to promote moldability and further enhance tribo‐positivity.^[^ ^120^ ^]^ The fabricated BW‐TENG, which was humidity‐ and wear‐resistant, environmentally friendly, and biodegradable, generated a high voltage of up to 3.2 kV in the rotation FS mode. A portable self‐powered agrosensing device was fabricated by combining the BW‐TENG with various circuits and units. This hybrid multichannel sensing system could detect variations in humidity, soil moisture, temperature, and light intensity and wirelessly transmit these data over a distance of up to 1.7 km (**Figure** **18**a). The circuit components for the entire system are shown in Figure 18b, and the wireless data transmission process is depicted in Figure 18c. As shown in Figure 18d, the 0.1 F supercapacitor used as an energy storage unit was charged to 5.07 V within 70 min. The appearance of the collected agrosensing data on the host computer is shown in Figure 18e.

Animal fur, which is a soft and elastic BW material, is highly suitable for preparing durable low‐wear rotary TENGs. A TENG was used to operate an environmental monitoring and automated irrigation system via energy harvested from flowing water and wind.^[^ ^16^ ^]^ The unit successfully functioned as a weather sensor by ringing an alarm during rainfall. Moreover, when attached to a water pump controller, the unit could effectively monitor the soil moisture level and efficiently activate the irrigation system.

Improving crop yields is a critical issue in crop cultivation.^[^ ^201^ ^]^ The application of an external voltage can improve plant growth and health by promoting plant metabolism, respiration, water absorption, and photosynthesis.^[^ ^202^, ^203^ ^]^ For this purpose TENGs can serve as voltage sources that generate high voltages from mechanical vibrations. Plant proteins, which can be isolated from agricultural and plant‐based waste, are widely available, inexpensive, and accessible. However, these materials are often neglected in the food industry because of their multiple isoionic points, complexity, antinutrients, undesirable taste, poor aqueous solubility, ionic strength, and temperature and pH sensitivity.^[^ ^204^, ^205^ ^]^ Plant proteins were recycled as BW‐TENG dielectrics that provided electric fields to stimulate crop growth, as shown schematically in Figure 18f.^[^ ^206^ ^]^ Two batches of bean sprouts were grown under the same environmental conditions, expect that one batch was subjected to an electric field produced by the BW‐TENG for a specific time interval. Notably, the sprouts grown under the applied electric field were healthier than those in the other batch (Figure 18g,h), possibly because improved water absorption in the presence of the electric field promoted seed incubation. Based on this success, a TENG‐operated setup for plant growth promotion was proposed for real‐life application (Figure 18i).

### 4.7 Air Cleaning

**Figure 19:**
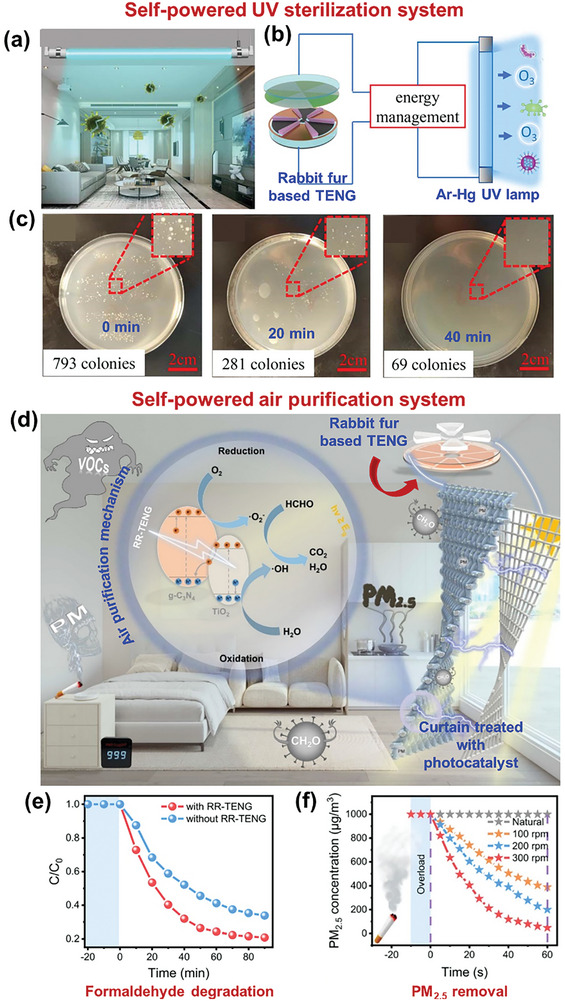
a) Schematic illustration of rabbit hair‐TENG‐operated self‐powered UV sterilization unit. b) Circuit diagram of the same. c) Photographs of *Escherichia coli* colonies sterilizing under the TENG powered UV irradiation for 0 min, 20 min, and 40 min. Reproduced with permission.^[^ ^32^ ^]^ Copyright 2023, IOP Publishing. d) Schematic illustration of the application scenario of the rabbit hair‐TENG powered curtain purification system for PM_2.5_ removal and formaldehyde degradation. SEM self‐powered smog removal system powered by TENG. e) Comparison of the formaldehyde degradation effect (with and without the TENG). f) Comparison of the PM_2.5_ removal at different rotation speed of the rotary TENG. Reproduced with permission.^[^ ^207^ ^]^ Copyright 2023, Royal Society of Chemistry.

Increasing air pollution and the presence of infectious bacteria or viruses in air require not only early detection methods but also effective disinfection/cleaning systems. TENGs can efficiently generate high voltages to drive sterilizing UV lamps. A rotary TENG in FS mode was fabricated using soft rabbit hair as a main component to achieve soft contact, thereby reducing mechanical abrasion and frictional heat to prolong the service life.^[^ ^32^ ^]^ The original DC output of the rotary TENG (≈1200 V) was modified using a voltage tripler (≈3440 V) and then used to drive a quartz glass‐tube UV lamp for self‐powered indoor air sterilization. A schematic of the self‐powered UV sterilization unit is shown in **Figure** **19**a, and the circuit schematics are shown in Figure 19b. The device generated ≈7 ppm ozone and could sterilize up to 91.3% of *Escherichia coli* cells within 40 min under UV irradiation (Figure 19c). An integrated curtain system for purifying indoor air was prepared using a rabbit‐fur‐based rotary TENG and a curtain sprayed with a g‐C_3_N_4_/TiO_2_ photocatalytic composite, as illustrated in Figure 19d.^[^ ^207^ ^]^ The strong electric field generated by the TENG enhanced formaldehyde and PM_2.5_ adsorption by the modified curtain (Figure 19e,f). In addition, the TENG‐generated electrostatic field improved photocatalytic formaldehyde degradation by the g‐C_3_N_4_/TiO_2_‐based curtain under illumination. The integrated unit removed ≈79.2% formaldehyde within 90 min and reduced PM_2.5_ from 999 to 50 µg m^−3^ within 1 min in an experimental chamber with an indoor‐like environment.

## 5 Challenges and Perspectives of BW‐TENGs

**Figure 20:**
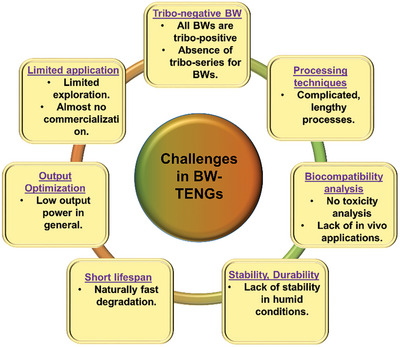
Schematic of the challenges occurring in BW‐TENGs.

Substantial advancements have been achieved in biocompatible and environment‐friendly TENGs using various types of BW, including untreated, treated, and carbonized plant‐ and animal‐based BW, as triboelectric materials. Numerous studies on the surface, structural, chemical, and mechanical properties, triboelectricity generation performance, biocompatibility, and water solubility of BW‐TENGs have led to improvements in functionality and applicability. In addition to being abundant, nontoxic, environmentally friendly, biocompatible, biodegradable, and accessible, BW materials are rich in electron‐donating functional groups and have high specific surface areas, which are crucial parameters for TENG operation. As revealed in Tables 2, 3, 4, the use of different types of BW materials to prepare TENGs has given rise to output voltages ranging from tens to hundreds of volts. Simply attaching a piece of dry leaf can generate a suitable voltage for operating small electronic devices,^[^ ^22^ ^]^ whereas utilizing rabbit fur, an easily obtainable pet shop waste, can produce an output voltage of thousands of volts while also with providing increased durability.^[^ ^32^ ^]^ Although significant progresses have been made in using this innovative ecofriendly approach to harness waste mechanical energy, various obstacles hinder the potential of BW‐TENGs and wide adoption for actual applications, as discussed below, which is summarized in **Figure** **20**. Till date, the BWs have been incorporated into TENGs by means of introducing biodegradability. In a practical point of view, the untreated BW‐TENGs are preferred for easy and cost‐effective fabrication approach. To liberate the constituent biopolymers from the unwanted substances, nature‐derived materials are chemically treated, and used for TENGs with/without other materials. For water‐resistant applications, the treated BWs are highly anticipated, as they can be chemically modified to reveal such water‐resistant characteristics. The carbonized BWs can be used to enhance triboelectric performance, thus developing flexible self‐powered devices.

### 5.1 Tribo‐Negative BW Materials

Almost all natural materials, including BW materials, are tribo‐positive owing to an abundance of electron‐donating functional groups, as discussed in Section 2.2. As tribo‐negative BW materials have never been investigated, synthetic polymers such as PDMS, PTFE, polyimide, and kapton are used as the tribo‐negative layers in most BW‐TENGs, which often limits their biocompatibility and applicability. When treated with chemicals such as nitrate complexes^[^ ^208^ ^]^ and triethoxy‐1*H*,1*H*,2*H*,2*H*‐tridecafluoro‐*n*‐octylsilane,^[^ ^209^ ^]^ CNFs exhibit tribo‐negative characteristics. Although these treatment processes rely on toxic chemicals, untreated lignocellulosic nanofibers derived from red cedar bark were recently reported to exhibit tribo‐negative characteristics.^[^ ^210^ ^]^ Further investigations are require to discover more inherently tribo‐negative BW materials. As an alternative approach, BW materials with weak electron‐donating characteristics can be paired with strongly tribo‐positive BW layers to prepare completely natural TENGs. For example, bacterial cellulose can be used for this purpose.^[^ ^211^ ^]^ Recently, two BW layers with unknown electronegativities were employed as opposite TENG layers. The electronegative and electropositive layers were differentiated empirically via analysis of the corresponding voltage peak patterns with a digital oscilloscope.^[^ ^136^ ^]^ Based on these findings, a series of triboelectric layers were classified in order of increasing electronegativity, as follows: eggshell membrane > dog hair > fish fins > tree cotton > PTFE. By expanding this approach to investigate other BW materials, a larger series of triboelectric BW materials could be identified, which would be extremely beneficial for preparing biocompatible BW‐TENGs.

### 5.2 Optimized Material Processing Techniques

The refinement of BW materials requires the complicated extraction and modification procedures, which are time‐consuming and often employ non‐ecofriendly chemicals. For example, the extraction of chitin, collagen, and gelatin requires chemical demineralization, whereas lignin and hemicellulose must be chemically extracted from plant‐based waste. The most common chemicals in these processes are HCl and NaOH, which are highly corrosive. Moreover, their use must be optimized for individual BW materials.^[^ ^212^, ^213^, ^214^ ^]^ to avoid degrading the morphology of the final material and thus affecting the triboelectric performance. To avoid the use of harmful chemicals, untreated BW materials are commonly used to prepare BW‐TENGs. As well as being simple and cost‐effective, this approach ensures that the unique microstructure of the BW material is retained. Unfortunately, there is little scope for optimizing TENG performance using current BW processing methods. Therefore, in most cases, the triboelectric output depends solely on the inherent properties of the BW material. To address this issue, the extraction processes should be further optimized. Furthermore, to improve TENG performance, low‐coat, simple, and sustainable methods should be developed for BW surface modification. Additionally, the integration of multiple BW materials into a single triboelectric layer should be investigated to determine whether this approach offers any advantages for the development of high‐performance TENGs.

### 5.3 Biocompatibility and Toxicity Analysis

Although BW and other biomaterials are typically considered to be biocompatible and environmentally friendly, some natural materials may contain residual hazardous compounds that could pose a serious threat.^[^ ^215^, ^216^ ^]^ Many biocompatible materials have shown impressive results in various implantable applications, including tissue remodeling,^[^ ^217^ ^]^ in vivo blood pressure monitoring,^[^ ^218^ ^]^ therapeutic simulation,^[^ ^219^ ^]^ and advanced neural interfaces.^[^ ^220^, ^221^ ^]^ The biodegradability and abundance of BW is expected to promote various self‐powered advanced applications. For example, BW‐TENGs have potential for use in e‐skin and wearable electronics. Moreover, BW‐TENGs may be suitable for implantable devices, although research in this area remains limited. Although soil decomposition tests and microbial tests have been used to examine the biodegradability and biocompatibility, respectively, of some BW‐TENGs,^[^ ^25^, ^35^ ^]^ these approach are insufficient for biomedical applications. The cytotoxicity and immunogenicity of BW‐TENGs should be studied extensively both in vivo and in vitro.^[^ ^6^ ^]^ The degradation of the constituent materials in vivo should also be carefully evaluated to determine how these materials break down and whether they are absorbed by or removed from the body.^[^ ^222^ ^]^ The long‐term effects of TENGs within the body should also be evaluated. After ensuring the safety of the active layers and other components in BW‐TENGs, the compliance of these materials with the standard guidelines for biomedical systems should be considered. Silk proteins from silkworms, which are natural biopolymers, have been widely used in drug delivery and tissue engineering applications.^[^ ^223^ ^]^ Furthermore, a recombinant spider‐silk‐based tribo‐/piezonanogenerator packaged ins a biocompatible silk‐based packet has been implanted in the chest of a Sprague Dawley rat to collect mechanical energy from the heartbeat.^[^ ^224^ ^]^ This device was suggested to be sufficiently safe for further in vivo applications. A completely biodegradable TENG prepared using commercially purchased cellulose, rice paper, chitin, egg white, and natural silk has been successfully applied for implantable in vivo applications.^[^ ^225^ ^]^ Moreover, these findings suggest that such materials can be directly extracted from natural sources for future biomedical applications. These studies can inspire similar initiatives with BW‐TENGs.

### 5.4 Stability and Durability in Humid Environments

Most BW materials are highly sensitive to humidity because they are highly hydrophilic or hygroscopic.^[^ ^226^ ^]^ Under humid conditions, water molecules readily enter the biomaterial matrix, which can significantly decrease the triboelectric output signal, thereby limiting the applicability of these materials in wearable electronics.^[^ ^192^ ^]^ Protracted exposure to humidity can deteriorate the energy conversion efficiency and functionality of TENGs. Owing to the humidity of the ambient atmosphere, the use of most BW‐TENGs is limited to a controlled laboratory environment. Hence, encapsulation or chemical treatment processes that provide hydrophobicity are critical. Chemical modification is an excellent approach for promoting hydrophobicity in natural materials. A piece of wood or other lignocellulosic materials can be made hydrophobic via treatment with tetramethylcyclotetrasiloxane^[^ ^227^ ^]^ or esterification.^[^ ^228^ ^]^ The hydrophobicity of other biomaterials can be enhanced using triethoxy‐1*H*,1*H*,2*H*,2*H*‐tridecafluoro‐*n*‐octylsilane (cellulose nanofibrils),^[^ ^209^ ^]^ by coupling alkyl chains with silane groups (collagen),^[^ ^229^ ^]^ of introducing acid anhydrides and acid chlorides (chitosan).^[^ ^230^ ^]^ To assemble a sustainable TENG, two fish‐scale‐derived gelatin films were subjected to different treatments.^[^ ^143^ ^]^ The first was one doped with dopamine (to serve as a tribo‐positive layer) and the second was treated with FOTS followed by oxygen plasma (to serve as the tribo‐negative layer.^[^ ^143^ ^]^ Owing to the extremely hydrophobic trifluoromethyl (–CF_3_) groups in FOTS, the FOTS‐modified gelatin film exhibited hydrophobic characteristics, as demonstrated by a water drop contact angle of 123°.

### 5.5 Fast Degradation and Short Lifespan

For environmental safety and sustainability, the biodegradability of BW‐based tribo‐active materials is advantageous; however, these characteristics can detrimentally affect long‐term stability and reusability. Consequently, BW‐based materials generally have a short lifespan.^[^ ^231^ ^]^ Plant‐based waste, such as leaves, petals, and fruit peels, dry gradually and lose their microstructure, resulting in a deterioration in triboelectric performance. Similarly, animal‐based BW materials, including fish scales, bladders, eggshell membranes, animal fur, and human hair, rapidly degrade in open environments, thereby impacting their TENG performance.^[^ ^194^ ^]^ To expand the service life of these materials, appropriate encapsulation methods must be considered. In this context, a biopolymer‐composite‐based TENG was prepared using a dry flower extract and PVA.^[^ ^25^ ^]^ Owing to the inclusion of PVA, this TENG generated a stable voltage of ≈80 V for 8 months.

### 5.6 Output Performance Optimization

In addition to capacitor charging and LED lightening, BW‐TENGs have been used as power sources in a variety of next‐generation applications. However, as the output voltage and power of most BW‐TENGs are relatively low, these systems can only operate small electronic devices. BW materials and their constituent biopolymers have inherent microstructures^[^ ^12^ ^]^ that are less optimizable than those of synthetic materials. This limitation can be overcome by combining multiple BW materials, adopting different device designs, or introducing other dielectric/conducting materials. BW materials have frequently been combined with other biopolymers and materials. For example, the triboelectric performance of eggshell membrane combined with commercial chitosan at different loadings has been investigated.^[^ ^95^ ^]^ The maximum output was achieved with 10% eggshell membrane. At higher loadings, the performance began to deteriorate, likely because of an uneven distribution or agglomeration in the composite. Furthermore, different types of textile waste, including biodegradable and semi‐/nonbiodegradable fibers, have combined to produce knitted TENGs.^[^ ^178^ ^]^ The triboelectric performance of these TENGs varied considerably owing to differences in surface morphology, but any other advantages or disadvantages of this approach have not been explored. BW active layers are sometimes combined with other inorganic compounds, either in the same layer^[^ ^62^ ^]^ or in the opposite layer,^[^ ^19^, ^195^, ^196^ ^]^ to introduce photosensitivity and produce BW‐TENG‐based self‐powered photodetectors. When discarded paper wipes were painted with carbon black, the charge density of the paper wipe increased from 0.4 to 35 µC m^−2^ and the output voltage of the corresponding TENG increased from a few volts to ≈80 V.^[^ ^165^ ^]^ In this system, the addition of carbon increased the effective surface area, which in turn enhanced triboelectrification. Moreover, the increased conductivity of the carbon‐coated triboelectric layer raised the charge‐transfer efficiency between the electrodes. Similarly, carbonized BW materials have been added as conducting materials to dielectric‐based triboelectric layers, which increased triboelectricity generation because the surface area was modified and enhanced charge transfer between the electrode pairs.^[^ ^62^ ^]^ Notably, by employing unique rotary structures, BW‐TENGs with higher output voltages (>1 kV) have been achieved.^[^ ^32^, ^77^, ^120^ ^]^ The incorporation of multiple BW materials into a single triboelectric layer has not yet been explored, and most of the other proposed solutions have not been thoroughly investigated; thus, various research directions are available for optimizing the output performance of BW‐TENGs.

### 5.7 Limited Exploration of Application Scope

To broaden the applicability of BW‐TENGs, their limited output voltage, poor stability, and low must be addressed. In particular, efforts should be focused on the further exploration and optimization of materials, structures, and innovative device designs as well as the development of appropriate circuit modules, depending on the application requirements. Furthermore, as BW‐TENGs are expected to be highly advantageous for implantable self‐powered biomedical devices, extensive biosafety evaluations are necessary.^[^ ^232^ ^]^ As evidenced by the applications reported to date, BW‐TENGs are highly sensitive to body motion, touch, and mechanical strain, making them suitable for HMI and robotic applications. By introducing water resistance into BW materials, the applicability of BW‐TENGs could be further expanded toward wearable sensors, energy harvesting from rain, and other water‐driven self‐powered applications via solid–liquid electrification.^[^ ^233^, ^234^ ^]^

## 6 Conclusion

This review provides a comprehensive analysis of the current state of BW‐TENG devices. Outstanding advances have been achieved in the development TENGs, and considerable progress has been made in the use of biocompatible materials for the fabrication of environment‐friendly TENGs. Notably, the recycling and upcycling of waste materials to fabricate TENGs contributes significantly to reducing the ecological impact of these devices.

Owing to their abundance and inherent triboelectric properties, BW materials have been extensively explored for TENG fabrication. Specifically, BW‐TENGs have been prepared using numerous plant‐ and animal‐based BW materials. A variety approaches has been used to incorporate BW materials into TENGs, including direct attachment, conversion into fine powders for either direct attachment or dispersion in a polymeric medium, chemical treatment to extract refined constituent materials, and carbonization. Despite being a relatively new approach, BW‐TENGs have been implemented in various modern applications. In particular, BW‐TENGs have been used to power small devices, light multiple LEDs, act as self‐powered sensors, develop HMI‐based devices, and monitor physiological signals.

Despite being sustainable and innovative approach, BW‐TENGs often suffer from certain limitations. Challenges such as the excessive use of chemicals during refinement, performance degradation due to humidity and other factors, short lifetimes, poor durability, and easy contamination must be addressed to broaden the commercial applicability of BW‐TENGs. In particular, the effects of mixing different BW materials on the stability, durability, and efficiency of TENGs should be explored.

Despite these challenges, BW‐TENGs are promising systems with wide applicability, especially for HMI devices, owing to their biocompatibility, portability, wearability, and efficiency. Following further investigation and the implementation of suitable material selection, processing, and device fabrication strategies, BW‐TENGs are expected to have a notable impact on next‐generation emerging bioelectronics while also contributing to environmental sustainability and pollution control.

## Author Contributions

A.B. performed conceptualization; investigation; and wrote the original draft. T.‐J.H. performed conceptualization; validation; supervision; wrote, reviewed and edited.

## Conflict of Interest

The authors declare no conflict of interest.
